# Bimetallic CuPd nanoparticles supported on ZnO or graphene for CO_2_ and CO conversion to methane and methanol†

**DOI:** 10.1039/d4su00339j

**Published:** 2024-09-04

**Authors:** Qaisar Maqbool, Klaus Dobrezberger, Julian Stropp, Martin Huber, Karl-Leopold Kontrus, Anna Aspalter, Julie Neuhauser, Thomas Schachinger, Stefan Löffler, Günther Rupprechter

**Affiliations:** a Institute of Materials Chemistry, TU Wien Getreidemarkt 9/BC 1060 Vienna Austria guenther.rupprechter@tuwien.ac.at; b University Service Center for Transmission Electron Microscopy, TU Wien Stadionallee 2/057-02 1020 Vienna Austria

## Abstract

Carbon dioxide (CO_2_) and carbon monoxide (CO) hydrogenation to methane (CH_4_) or methanol (MeOH) is a promising pathway to reduce CO_2_ emissions and to mitigate dependence on rapidly depleting fossil fuels. Along these lines, a series of catalysts comprising copper (Cu) or palladium (Pd) nanoparticles (NPs) supported on zinc oxide (ZnO) as well as bimetallic CuPd NPs supported on ZnO or graphene were synthesized *via* various methodologies. The prepared catalysts underwent comprehensive characterization *via* high-resolution transmission electron microscopy (HRTEM), energy-dispersive X-ray spectroscopy (EDX) mapping, electron energy loss spectroscopy (EELS), X-ray diffraction (XRD), hydrogen temperature-programmed reduction and desorption (H_2_-TPR and H_2_-TPD), and deuterium temperature-programmed desorption (D_2_O-TPD). In the CO_2_ hydrogenation process carried out at 20 bar and elevated temperatures (300 to 500 °C), Cu, Pd, and CuPd NPs (<5 wt% loading) supported on ZnO or graphene predominantly yielded CH_4_ as the primary product, with CO generated as a byproduct *via* the reverse water gas shift (RWGS) reaction. For CO hydrogenation between 400 and 500 °C, the CO conversion was at least 40% higher than the CO_2_ conversion, with CH_4_ and CO_2_ identified as the main products, the latter from water gas shift. Employing 90 wt% Cu on ZnO led to an enhanced CO conversion of 14%, with the MeOH yield reaching 10% and the CO_2_ yield reaching 4.3% at 230 °C. Overall, the results demonstrate that lower Cu/Pd loading (<5 wt%) supported on ZnO/graphene favored CH_4_ production, while higher Cu content (90 wt%) promoted MeOH production, for both CO_2_ and CO hydrogenation at high pressure.

## Abstract 2

CO_2_ and CO valorization to methanol and methane over Cu or CuPd nanoparticles supported on ZnO or graphene. The catalysts demonstrate high efficiency, favouring methane at lower metal loading but methanol at high copper content.

## Graphical abstract

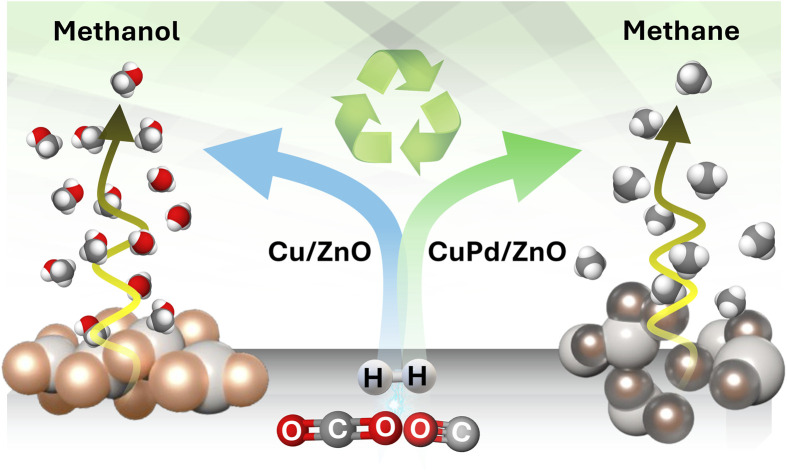

**Sustainability spotlight**

The hydrogenation of carbon dioxide (CO_2_) to methane (CH_4_) or methanol (CH_3_OH) is a promising route to use CO_2_ as feedstock, lowering its emissions and reducing dependence on fossil fuels. It aligns well with the UN's Sustainable Development Goals on energy, sustainability and climate. This study reports the synthesis of copper-based catalysts supported on zinc oxide or graphene, promoted by palladium. The nanoparticle catalysts demonstrate high efficiency in CO_2_ and CO hydrogenation, favouring methane production at lower metal loadings but methanol production at higher copper content. This sustainable approach highlights the potential for reducing environmental impact by improving catalytic performance.

## Introduction

Carbon dioxide (CO_2_) is Earth's most abundant greenhouse gas responsible for absorbing and radiating heat.^1^ As of April 2024, the atmospheric CO_2_ concentration observed by the NOAA Global Monitoring Lab reached 425.38 ppm, marking a new record.^2^ This value surpasses any previous levels observed in human history and it is expected to increase to ≈500 ppm in the next 20 years, which is regarded as the point of no return.^3^ Accordingly, the International Energy Agency (IEA) took the initiative of Carbon Capture, Utilization and Storage (CCUS)^4^ and Net Zero Emissions by the 2050 (NZE)^5^ Scenario. However, despite the existence of numerous strategies, effective solutions to this challenge remain elusive.

The increase in CO_2_ concentration is largely attributed to burning fossil fuels for energy generation and use as feedstock for chemicals. The utilization of CO_2_ as an alternative and sustainable carbon source has thus gained worldwide attention as a key aspect of CCUS.^6^ The advancements in energy-efficient catalytic CO_2_ conversion using renewable energy have the potential to mitigate CO_2_ emissions and reduce reliance on fossil fuels.^7^ One of the straightforward ways to utilize CO_2_ is its hydrogenation to produce value-added products such as hydrocarbons (including olefins, liquid hydrocarbons, and aromatics), oxygenates (such as alcohols and dimethyl ether) or reaction gases such as CO (for syngas and Fischer–Tropsch processes) or methane.^8,9^ The three primary catalytic methods for CO_2_ hydrogenation include thermal catalysis,^8–20^ electrocatalysis,^21,22^ and photocatalysis.^23–26^ Although thermal catalysis requires higher temperatures and pressures for CO_2_ activation, it remains a widely applied process for rapid and efficient transformation of CO_2_ (that could originate from carbon capture).

Due to thermodynamic limitations, hydrogenation to methanol (MeOH) demands high-pressure and elevated operating temperature to achieve favorable reaction kinetics, as well as an effective and active catalyst. In this regard, various catalytic materials including Pt, In, Ni, Ru, Zr, Rh, and Ga have been widely explored for CO_2_ hydrogenation to MeOH.^27–32^ However, Cu still stands out as the most utilized and studied metal^16–18^ due to its high activity at lower temperatures, making it particularly suitable for the CO_2_ hydrogenation reaction. Additionally, Cu offers advantages in terms of cost-effectiveness, as well as its multivalency (Cu^0^, Cu^I^, and Cu^II^).^19^ Moreover, Cu maintains stable interaction with oxygen, preventing the formation of unstable intermediates or surface poisoning. The CO-induced mobility of Cu atoms may counteract sintering.^33,34^ It also exhibits strong interactions with other materials, such as Zn, which improves stability, selectivity, and the ability to dissociate H_2_.^35,36^

Industrially, MeOH is mainly synthesized from a feed of CO_2_, CO, and H_2_ at high pressure (100 bar), using Cu/ZnO/Al_2_O_3_ catalysts.^37,38^ The kinetics and thermodynamics of this reaction have been widely studied^11,39–41^ along with the influence of water on catalyst performance.^42–44^ There are three main reactions involved in the hydrogenation of CO_2_,

Reverse water gas shift (RWGS):CO_2_ + H_2_ → CO + H_2_O Δ*H*_25°C_ = 41 kJ mol^−1^ (ref. **8**)

Methanation:CO_2_ + 4H_2_ → CH_4_ + 2H_2_O Δ*H*_25°C_ = −164 kJ mol^−1^ (ref. **45**)

MeOH synthesis:CO_2_ + 3H_2_ → CH_3_OH + H_2_O Δ*H*_25°C_ = −49.5 kJ mol^−1^ (ref. **46**)

The reaction selectivity is thus influenced by the reaction temperature, pressure, and the catalyst used. Additionally, the catalytic performance of Cu can be further improved by the addition of promoters (*e.g.* Mn, Zr, Zn, and Al)^47,48^ and by various supports (*e.g.* graphitic carbon, SiO_2_, CNTs, and MgO).^49–52^ This can modulate active sites and the surface charge of Cu.^53^

Studies have shown that the use of Pd, either alone or as a promoter of Cu, both over ZnO, can enhance selectivity towards various products of CO_2_ hydrogenation.^54,55^ Siriworarat *et al.*^56^ investigated the impact of Pd loading (5, 10, and 15 wt%) on a Pd–Cu–Zn catalyst, where Cu and Zn comprised 25 wt%. Conducted at 250 °C and 25 bar, the study demonstrated that the highest performance was achieved with the catalyst containing 15 wt% Pd, 25 wt% Cu, and 25 wt% Zn, yielding a MeOH space-time yield of 0.112 kg_MeOH_ kg_cat_^−1^ h^−1^. In another study by Díez-Ramírez *et al.,*^57^ 37.5 wt% Pd with CuZn/SiC exhibited the highest selectivity towards MeOH. In addition, Deerattrakul *et al.*^7^ found that 15 wt% CuZn(O) loading over a nitrogen-doped graphene aerogel achieved the highest MeOH production (0.095 kg_MeOH_ kg_cat_^−1^ h^−1^) at a reaction temperature of 250 °C and a relatively low pressure of 15 bar. Collectively, the combination of Cu with Pd over ZnO or graphene as the support may present an intriguing avenue to enhance CO_2_ hydrogenation to hydrocarbons, warranting further exploration through studies of monometallic Cu/ZnO, bimetallic CuPd/ZnO, or CuPd/Gr. Even more so, as one would rather avoid using noble metals.

To add to the current understanding, different preparation protocols were adapted to prepare ZnO-supported CuPd (bimetallic) and graphene-supported CuPd, as well as ZnO supported Cu or Pd (monometallic) catalysts, as summarized in Table 1. The reduction temperature of the catalysts (monometallic) was varied (*i.e.*, 200, 300, and 500 °C) to investigate its influence on catalytic activity, catalyst morphology, particle size, and composition. High resolution transmission electron microscopy (HRTEM) with electron energy loss spectroscopy (EELS) and energy-dispersive X-ray spectroscopy (EDX), high-angle annular dark-field scanning transmission electron microscopy (HAADF-STEM), and X-ray powder diffraction (XRD) were employed to analyze the size, distribution of metal particles, and the catalysts' composition. Furthermore, temperature-programmed reduction (TPR) provided insights into catalyst stability and metal–support interactions. Moreover, temperature-programmed desorption (TPD) revealed information about adsorption sites. On the reduced catalysts, the hydrogenation of CO_2_ was carried out at atmospheric pressure and an elevated pressure of 20 bar in a continuous flow fixed-bed reactor, connected to a micro-gas chromatograph (GC) for kinetic measurements, with the reaction conducted at different temperatures. Selectivity and catalytic activity as a function of reaction temperature were compared for each catalyst.

**Table 1:** Catalysts examined in the current study (% refers to wt%)

Synthesized catalysts	Abbreviation
5% CuPd (3 : 2) on ZnO or graphene (reduction method)	(i) CuPd/ZnO-r
	(ii) CuPd/Gr-r
5% CuPd (3 : 2) on ZnO (polyol synthesis method)	(iii) CuPd/ZnO-p
90% Cu- and Zn-nitrates reduced with Na_2_CO_3_ (co-precipitation method)	(iv) 90Cu/ZnO-c
5% Cu or Pd on ZnO (wet impregnation method)	(v) Cu/ZnO-i
	(vi) Pd/ZnO-i

## Results and discussion

### Catalyst synthesis and characterization

**Fig. 1:**
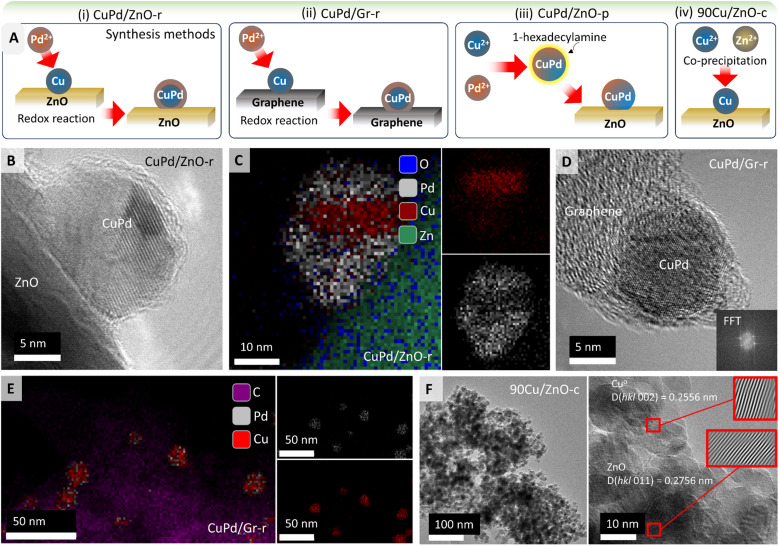
(A) Schematics elaborating the synthesis of nanocatalysts through different methods, mainly (i) 5%Cu/Pd (3 : 2) over ZnO or (ii) graphene through the liquid reduction method, (iii) Pd^2+^ and Cu^2+^ reduction with ethylene glycol, stabilization with 1-hexadecylamine and deposition on ZnO to obtain CuPd/ZnO-p, and (iv) 90% Cu with Zn nitrates, reduced with aqueous Na_2_CO_3_ to produce 90Cu/ZnO-c (for the details of synthesis see the Experimental section), (B) HRTEM image of the bimetallic CuPd/ZnO-r catalysts, (C) corresponding EDX mapping showing the distribution of ‘Pd’, ‘Cu’ and ‘O’ atoms in CuPd/ZnO-r, (D) HRTEM image of the CuPd/Gr-r catalysts, (E) EDX mapping showing the distribution of ‘C’, ‘Pd’, and ‘Cu’ atoms in CuPd/Gr-r and (F) HRTEM image of the 90Cu/ZnO-c catalysts, also showing the interplanar spacing of Cu (*d* = 0.256 nm) and ZnO (*d* = 0.277 nm).

Fig. 1A illustrates the synthesis procedures for obtaining different types of catalysts using ZnO or graphene (Gr) as supports (Table 1; for details see the Experimental section). Following a redox method (denoted by r)^58^ for 5 wt% Cu/Pd (3 : 2), H_2_-reduced Cu on ZnO or Gr was mixed with Pd salt under N_2_ (Cu + Pd^2+^ → Cu^2+^ + Pd) forming (i) CuPd/ZnO-r or (ii) CuPd/Gr-r catalysts, respectively.

In a polyol synthesis,^59,60^ Pd^2+^ and Cu^2+^ ions were reduced with ethylene glycol and stabilized with 1-hexadecylamine as a ligand. The obtained CuPd alloy nanoparticles (NPs) were impregnated on the ZnO support forming (iii) CuPd/ZnO-p (p denotes polyol synthesis). Moreover, adopting a co-precipitation method,^61^ 90 wt% Cu and Zn nitrates were reduced together with aqueous Na_2_CO_3_, dried, and calcined at 350 °C to obtain (iv) the 90Cu/ZnO-c catalyst (c denotes co-precipitation). (v) Cu/ZnO-i and (vi) Pd/ZnO-i were prepared by wet impregnation (denoted by i). In the following, for simplicity %loading refers to wt%.

In order to determine the morphology, elemental composition, and metal–support interactions of the prepared catalysts, HRTEM, EDX, and EELS mapping were employed. CuPd/ZnO-r was examined after H_2_ pretreatment (Fig. 1B) and after CO_2_ hydrogenation, as shown in Fig. 1C and S1.† A notable aspect (EDX mapping in Fig. 1C) is the segregation of Cu and Pd within the NPs. Two potential reasons may account for CuPd/ZnO-r possessing a core–shell structure, formed during synthesis. As shown in Fig. 1A, CuPd/ZnO-r is formed through the direct deposition of Pd *via* a redox reaction, with Cu/ZnO already in place while Pd coats the Cu, resulting in the development of layered NPs. Second, the layered structure may arise from reaction conditions, as CO can displace (mobilize) Cu atoms,^33,62^ while the strong binding of CO to Pd drives its surface segregation. Given the layered morphology of the NP, the Cu : Pd ratio does not correspond to a specific phase. The line scan of a NP after CO hydrogenation (Fig. S2†) confirmed that Cu is predominantly present in the core, while Pd is mainly located on the outer shell, which is also consistent with CuPd/ZnO-r observed after CO_2_ hydrogenation (Fig. S1†). Furthermore, the concentration of Zn within the particle increases in the vicinity of the ZnO support. Since there is no concurrent increase in oxygen within the particle, it suggests that Zn interacts with the particle, forming an interface alloy between the particle and support.

The bimetallic CuPd/ZnO-p catalyst was examined both before (Fig. S3†) and after CO_2_ hydrogenation (Fig. S4†). The EDX maps in Fig. S3† suggest that the NPs were CuPd alloys, with Fig. S4† showing a nearly uniform distribution of Cu and Pd within the NPs. No metallic Zn was detected in the NPs, although XRD detected PdZn alloys (see below).

A HRTEM image of the reduced CuPd/Gr-r catalyst is presented in Fig. 1D. By performing fast Fourier transformation (FFT) of a small image area encompassing the NP, the lattice distance was determined. This analysis was conducted for four different NPs, yielding the results listed in Table S1.† The percentage of Cu was calculated using Vegard's rule, which assumes that an arbitrary mixture of two components (such as Cu and Pd) has the same face-centered cubic (fcc) crystal structure. Based on the lattice constant of Cu of 0.361 nm and that of Pd of 0.389 nm, successful incorporation of Cu into the Pd lattice can be assumed. Additionally, EDX analysis was applied for determining the elemental composition (Fig. 1E). EDX phase mapping clearly shows that the NPs mainly consist of Cu and Pd as assumed for Vegard's rule. The accompanying EDX plot (Fig. S5†) shows the quantification spectra of the NPs, with results listed in Table S2.† Similar to the results obtained *via* Vegard's rule, no discernible trend regarding the NP composition (Cu/Pd) ratio was observed.

In addition to EDX mapping, EDX line scans of the CuPd/Gr-r catalyst were acquired, depicted in Fig. S6.† Notably, the NP in the central image exhibited a much lower Pd percentage than the other NPs. In the left image, an apparent increase in carbon content inside the NP was observed nearer to the support. Given that the inclusion of carbon inside the NPs is unlikely, the signal rather originates from the graphene support under the NP. The line scan in the right image traverses two NPs, with Cu found between them, but no Pd. This suggests that sintering is initiated by the movement of Cu atoms,^33^ leading to coalescence on the graphene support.

The catalyst obtained *via* the co-precipitation method, with 90% Cu over ZnO (90Cu/ZnO-c), exhibited an average particle size of 12 nm (Fig. 1F), with NPs uniformly dispersed. The interplanar spacing of Cu was *d* = 0.256 nm, while for ZnO it was *d* = 0.277 nm. The lattice constant of fcc Cu is 0.361 nm, while ZnO exhibits a hexagonal crystal structure, with lattice parameters *a* = 0.32496 nm and *c* = 0.52042 nm. This confirms the assignment of the interplanar spacings and lattice structures to Cu and ZnO.

Similarly, the catalysts with low Cu or Pd loading (5%) over ZnO, obtained through a one pot wet-impregnation method, were also analyzed. The Cu/ZnO-i catalyst after reduction in H_2_ showed a single Cu NP on ZnO (Fig. S7†). Benefitting from the high resolution, lattice planes were analyzed for phase identification, *i.e.* in the red square an FFT was generated. After inverse FFT, the image revealed individual lattice planes without interference from background signals. The distance between lattice planes was measured to be 0.251 nm. Fig. S7† illustrates the unit cell of Cu, focusing on (1 1 0) lattice planes marked in purple, where the distance between two Cu atoms was measured as *d*(Cu–Cu) = 0.25 nm, as observed in the HRTEM image.

Additionally, Fig. S8† depicts a high-angle annular dark-field scanning transmission electron (HAADF-STEM) micrograph of the Pd/ZnO-i catalyst after H_2_ reduction at 500 °C. Variations in intensity in HAADF images are attributed to differences in sample thickness or the atomic number. The red box surrounding the NP on the surface of the ZnO support (Fig. S8†) was chosen for EDX analysis revealing reduced Pd and Zn, with an overall composition of 46.6 at% Pd and 53.4 at% Zn.

Fig. S9 and S10† reveal the effect of high pressure (20 bar) and temperature (500 °C) on NP size in the course of the hydrogenation reaction. Bimetallic NPs (CuPd) showed significantly more sintering on graphene, increasing in size from 8.7 nm to 20 nm, than when supported on ZnO, for which the NP size only increased from 8.4 nm to 11 nm. In contrast, monometallic NPs of both Cu and Pd on ZnO even exhibited a slight reduction in size, likely due to CO-induced redispersion (Fig. S9†).

**Fig. 2:**
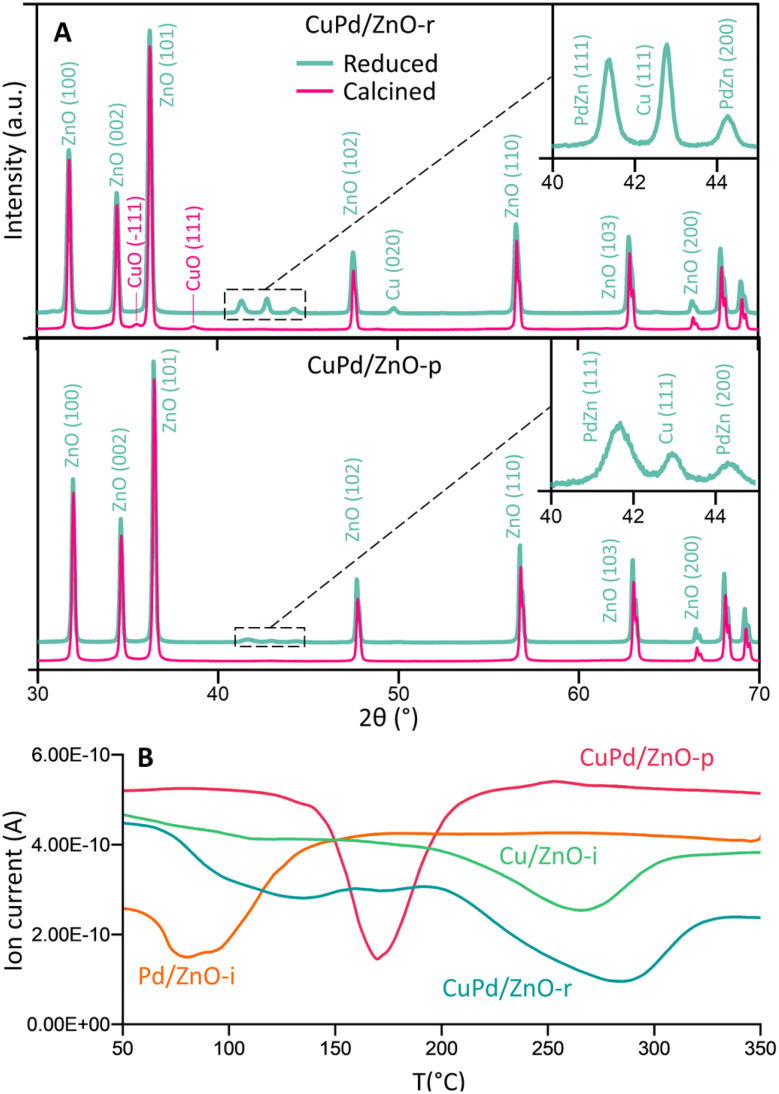
(A) Crystal structure of calcined (500 °C) and H_2_-reduced CuPd/ZnO-r and CuPd/ZnO-p catalysts. The crystal structure of other catalysts can be seen in Fig. S9–S11.† (B) H_2_-temperature programmed reduction (TPR) profile of calcined (500 °C) CuPd/ZnO-r, CuPd/ZnO-p, Cu/ZnO-i and Pd/ZnO-i catalysts.

Fig. 2A and S11–S13† display the (area-averaged) X-ray diffraction (XRD) patterns of the as-prepared (calcined) and H_2_-reduced catalysts. For the CuPd/ZnO-r catalyst in the calcined state, CuO was identified, with no mixed CuPd oxide detected. Due to the small size of the metal particles, the Bragg peaks appear broader and blend into the background noise. Pd exhibits a higher affinity towards Zn, as a separate PdZn (1 1 1, 2 0 0) phase was detected after H_2_ reduction at 500 °C, still with no signs of a CuPd alloy. This suggests that in CuPd/ZnO, Cu and Pd separate, also forming PdZn interfaces, in line with the locally resolved HRTEM studies (see Fig. 1B). The CuPd/ZnO-p catalyst showed similar XRD patterns before and after H_2_ reduction, with peaks related to PdZn being less intense than the ones for CuPd/ZnO-r. Conversely, the CuPd/Gr-r catalyst (Fig. S11†) after H_2_ reduction exhibited dominant diffraction from graphitic carbon (0 0 2), because of graphene calcination at elevated temperatures, in line with literature reports.^63,64^ Furthermore, the XRD patterns of the Cu/ZnO-i and Pd/ZnO-i catalysts (Fig. S12 and S13†) after pretreatment in H_2_ at different temperatures confirmed that PdO was reduced at 200 °C, while CuO reduction occurred at a higher temperature of 300 °C.

Temperature-programmed reduction (TPR) studies were thus carried out to monitor the H_2_-induced reduction behaviour of the (calcined) catalysts in more detail (10% H_2_ in Ar). The mass spectrometer (MS) signals of H_2_ (*m*/*z* = 2) were plotted against temperature, as shown in Fig. 2B and S14.† Pd/ZnO-i exhibited H_2_ consumption starting at a minimum temperature of 80 °C, while Cu/ZnO-i was reduced at 270 °C. In contrast, CuPd/ZnO-r and CuPd/ZnO-p displayed distinctly different profiles. For CuPd/ZnO-r, the minimum of Pd was slightly shifted to higher temperature (130 °C), while the minimum for Cu was at 290 °C. This again suggests that CuPd/ZnO-r consists of somewhat separate, but still connected Cu and Pd, likely within the NPs. The CuPd/ZnO-p profile exhibited only a single minimum at 170 °C, indicating the presence of alloyed CuPd NPs.

**Fig. 3:**
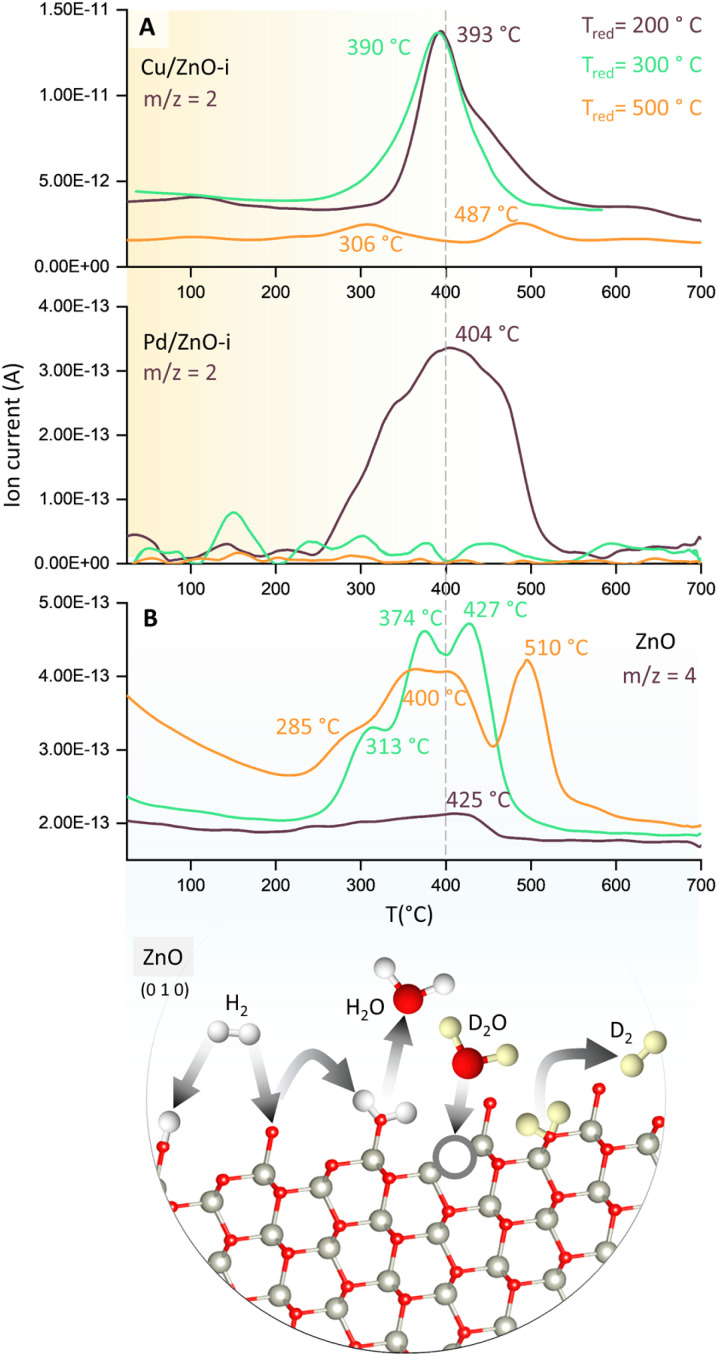
Temperature programmed desorption (TPD) studies of catalysts. (A) H_2_-TPD of Cu/ZnO-i and Pd/ZnO-i for different reduction temperatures with *m*/*z* = 2 recorded and (B) D_2_O-TPD of ZnO for different reduction temperatures with *m*/*z* = 4 recorded. Structure of ZnO with possible reaction pathways of H_2_ and D_2_(O) on the ZnO surface.^65^

To evaluate the binding strength and distribution of H_2_ adsorption sites, hydrogen-temperature programmed desorption (H_2_-TPD) was performed, which also shed light on metal–support interactions. H_2_-TPD was performed for both catalysts (Cu and Pd/ZnO-i) for each reduction temperature (*T*_200_, *T*_300_, and *T*_500°C_). In Fig. 3A, the H_2_ desorption profile of the Cu/ZnO-i catalysts revealed desorption maxima at 390 and 393 °C for 200 and 300 °C reduction temperatures, respectively. In contrast, the Cu/ZnO-i catalyst reduced at 500 °C did not exhibit a clear desorption peak, potentially due to the high-temperature reduction process leading to the formation of a CuZn alloy or sintering, which weakens the chemical bonding of H_2_ on the catalyst surface. Similarly, at higher reduction temperatures (*T*_300_ and *T*_500°C_), the Pd/ZnO-i catalysts did not display the desorption maximum observed after *T*_200°C_ (Fig. 3A) anymore. This again suggests that Pd can rather easily form an alloy with Zn at temperatures exceeding 200 °C.^66–68^ Metallic Pd can absorb hydrogen into the NPs, which ceases upon forming a PdZn alloy. Analogously, previous studies with Pd–Pb alloys indicated a decreasing intensity of H_2_ desorption upon addition of Pb.^69^ For the calculation of the binding energy of molecules on the catalyst's surface, the Redhead approximation was applied.^70^ The energy of desorption (*E*_des_) for Cu/ZnO-i was *E*_des_ = 169 kJ mol^−1^ at *T*_max_ = 390 °C, while for Pd/ZnO-i, it was *E*_des_ = 174 kJ mol^−1^ at *T*_max_ = 410 °C.

Furthermore, to detect surface defects in the ZnO support,^71^ TPD was performed with deuterium oxide (D_2_O-TPD). The desorption behaviour of D_2_ from D_2_O adsorbed on the catalyst surface is indicative of the nature and density of surface defects or oxygen vacancies.^72^ Specifically, the desorption temperature and intensity of D_2_ desorption peaks can reveal the availability and accessibility of surface defect sites. Higher desorption temperatures and/or broader desorption peaks may suggest stronger interactions between D_2_O and defects, indicating vacancies in the ZnO support, which can influence the CO_2_ and CO hydrogenation reactions.^73^

In Fig. 3B, D_2_ desorption from the ZnO surface is displayed. After catalyst reduction at 200 °C and D_2_O dosing, only weak D_2_ desorption was observed. However, D_2_ desorption peaks were clearly visible for reduction at 300 and 500 °C, with peak maxima ranging from 313 to 427 °C and 285 to 510 °C, respectively. A desorption peak at approximately 370 °C is evident for every reduction temperature. Fig. 3B also illustrates potential processes involving the interaction of H_2_ and H_2_O, or D_2_ and D_2_O, with O vacancies on the ZnO surface. Due to the interaction of D_2_O with the surface defects of ZnO, oxygen vacancies can be re-filled and D_2_ formed (*m*/*z* = 4). The literature also reported the observations of surface defects in hydrothermally grown ZnO after exposure to D_2_ gas at elevated temperatures.^74^

### CO_2_ and CO hydrogenation to methane or methanol

**Fig. 4:**
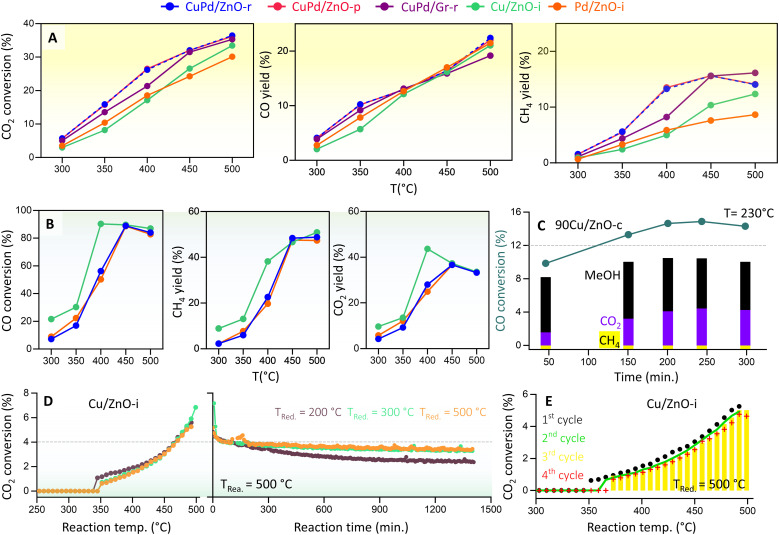
Kinetic measurements of CO_2_ and CO hydrogenation. (A) Catalytic performance of CuPd/ZnO-r, CuPd/ZnO-p, CuPd/Gr-r, Cu/ZnO-i, and Pd/ZnO-i catalysts in terms of CO_2_ conversion and CO and CH_4_ yields at *p* = 20 bar and *T* = 300–500 °C, and (B) CO conversion and CH_4_ and CO_2_ yields for CuPd/ZnO-r, Cu/ZnO-i, and Pd/ZnO-i at *p* = 20 bar and *T* = 300–500 °C. (C) CO hydrogenation to MeOH (%) and CO_2_ (%) by 90Cu/ZnO-c catalysts at *p* = 20 bar and *T* = 230 °C (for CO_2_ hydrogenation, see Fig. S15†), (D) CO_2_ conversion (%) to CO by Cu/ZnO-i catalysts reduced at different temperatures, at *p* = 1 bar and *T* = 300–500 °C, and “reaction time” refers to the time on stream under constant conditions (for the Pd/ZnO-i catalyst, see Fig. S17†), and (E) CO_2_ conversion (%) to CO over multiple cycles (for the Cu/ZnO-i catalyst reduced at 500 °C).

We then determined the CO_2_ hydrogenation performance of the various catalysts (Fig. 4A) at 20 bar pressure and temperatures from 300 to 500 °C, including CuPd/ZnO-r, CuPd/ZnO-p, CuPd/Gr-r, 90Cu/ZnO-c, Cu/ZnO-i, and Pd/ZnO-i. Prior to each measurement, the catalysts were reduced in a hydrogen flow. The reaction mixture consisted of 5% CO_2_ and 20% H_2_ in helium. CO_2_ conversion increased with temperature, rising from about 5% at 300 °C to >30% at 500 °C. Notably, for the bimetallic catalysts, CO_2_ conversion was at least 3% higher across all measured temperatures. CO and CH_4_ were detected as the main products of CO_2_ hydrogenation, with the CO yield increasing linearly with temperature, reaching 20% at 500 °C. For both CuPd/ZnO-r and CuPd/ZnO-p, a significant increase was also observed in CH_4_ yield (from 5.8% to 13%) as the temperature increased from 350 to 400 °C. At 500 °C, CuPd/ZnO-r exhibited the highest CH_4_ yield (16%) as compared to CuPd/ZnO-p and CuPd/Gr, which both yielded 14%. The monometallic Cu/ZnO-i had a CH_4_ yield of 12%, while Pd/ZnO-i showed the lowest yield of 8%.

Very differently, the catalyst containing 90 wt% Cu (90Cu/ZnO-c), prepared by co-precipitation and tested at 230 °C, exhibited selectivity towards MeOH rather than CH_4_ (Fig. S15†). Despite a CO_2_ conversion of 5.5%, the MeOH yield remained constant over time, with little CH_4_ detected. Overall, the CO yield was 4%, while MeOH was recorded at 2%. A similar trend had been observed in previous studies, demonstrating improved MeOH selectivity/yield upon high Cu loading on ZnO^10,75^ and observation of a volcano-like pattern in MeOH selectivity with respect to the Cu NP size.^76^

Furthermore, as RWGS may be followed by CO hydrogenation, the latter reaction was evaluated on Cu/ZnO-i, Pd/ZnO-i, and CuPd/ZnO-r under conditions similar to those of CO_2_ hydrogenation, and only 5% CO was used instead of CO_2_ (Fig. 4B). Notably, from 400 to 500 °C, CO conversion was at least 40% higher than CO_2_ conversion. CH_4_ (from methanation) and CO_2_ (from WGS) were identified as the main products of CO hydrogenation, with the CO_2_ yield increasing linearly with temperature, peaking at 37% at 450 °C and then dropping to 33% at 500 °C. The CH_4_ yield reached its highest level of 48% at 450 °C and remained steady up to 500 °C. No significant differences were observed between monometallic or bimetallic catalysts for CO hydrogenation. Nevertheless, ethane (from FT) was detected as a side product (≈4%) at 400 °C (Fig. S16†). Interestingly, the 90Cu/ZnO-c catalyst showed a higher CO conversion of 14%, with a MeOH yield of 10% and a CO_2_ yield of 4.3% at 230 °C (Fig. 4C). Overall, the MeOH yield remained constant at 8–10% over 300 minutes. This further confirmed that a CO/H_2_ gas feed increases the MeOH yield (CO + 2H_2_ → CH_3_OH).^20,41^ Indeed, for the best performance, a mixture of CO_2_/CO/H_2_ (5%/20%/75%) is known to be required.^77^ The conducted experiments confirm that the hydrogenation of CO yields more CH_4_ or MeOH than CO_2_ hydrogenation. Thus, for MeOH production at the industrial level using Cu/ZnO-based catalysts, CO serves as the major feed gas (20%) alongside CO_2_ and H_2_.^14^

The catalytic performance of the best performing catalysts was further described by calculating the space-time yield (STY) (more details in the ESI†), which includes the amount of the catalyst (0.1 g) used during the reaction:

• For CO_2_ hydrogenation to CH_3_OH over 90Cu/ZnO-c, the STY was 0.0836 g_MeOH_ g_cat_^−1^ h^−1^, and for CO hydrogenation, its STY was 0.4811 g_MeOH_ g_cat_^−1^ h^−1^.

• For CO_2_ hydrogenation to CH_4_, CuPd/Gr-r showed the best performance with a STY of 0.2416 g_CH_4__ g_cat_^−1^ h^−1^, while for CO hydrogenation, Cu–ZnO-i exhibited the best STY of 0.7618 g_CH_4__ g_cat_^−1^ h^−1^.

A CuO/ZnO catalyst obtained through an ammonia-evaporation-induced synthetic method involving the impregnation of filament-like ZnO for CO_2_ hydrogenation at 3 bars showed the best performance, with a MeOH STY of 0.55 g_MeOH_ g_cat_^−1^ h^−1^ and a selectivity of 78.2%.^78^ Moreover, Han *et al.*^79^ observed that doping Cu/ZnO catalysts with Pd, Pt, and Ru led to the formation of electron-rich Cu sites due to partial electron transfer, greatly enhancing CO_2_ activation. The Pd-doped catalyst demonstrated the highest MeOH STY of 0.52 g_MeOH_ g_cat_^−1^ h^−1^ with 100 hours of stability. In contrast, over a Cu_0.5_Ni_0.5_/γ-Al_2_O_3_ catalyst at 5 bar pressure, Reddy *et al.*^80^ reported CO_2_ conversion not exceeding 30% to CO. During the reaction, the Cu core atoms migrated toward the surface, resulting in the restructuring of the Cu@Ni core–shell structure to a Cu–Ni alloy, which functioned as the active site by enhancing CO desorption.

To examine the effect of pressure, CO_2_ hydrogenation was also studied at atmospheric pressure (1 bar) from 250 to 500 °C, using Cu/ZnO-i or Pd/ZnO-i catalysts, pre-reduced at different temperatures (*T*_red_ = 200, 300, and 500 °C) (Fig. 4D). CO was the primary product of both catalysts. The CO_2_ conversion of Pd/ZnO-i was 11% higher than that of Cu/ZnO-i for every reduction temperature. A reaction temperature of about 300 °C was necessary for CO formation with the Pd/ZnO-i catalyst, whereas with Cu/ZnO-i amounts of CO were first detected at 380 °C. According to the literature,^81–84^ the reaction mechanism involves the adsorption of CO_2_ on the ZnO-supported Cu or Pd catalysts, leading to intermediate-carbonate formation.

To evaluate the catalysts' stability, a 24 hour reaction was carried out at 500 °C. Each catalyst underwent reduction pretreatment at 200, 300, or 500 °C before the long-term reaction. The Cu/ZnO-i catalyst reduced at 200 °C exhibited more pronounced deactivation than those pre-reduced at 300 or 500 °C (Fig. 4D). It appears that reduction at 200 °C was insufficient, with CuO still being present. During the reaction, the partly reduced sample was subjected to 500 °C and a gas flow of 20 vol% H_2_, leading to sintering of the remaining CuO NPs and final reduction to metallic Cu. The Cu/ZnO-i catalysts reduced at 300 and 500 °C displayed almost constant CO_2_ conversion to CO over the 24 hour reaction period (Fig. 4D). In contrast, the Pd/ZnO-i catalyst exhibited some CO_2_ conversion to CO at 200 °C, possibly due to metallic Pd already being present on the catalyst surface at this reduction temperature (Fig. S15†). The increase in reduction temperature resulted in surface alloy formation,^55,85^ as indicated by the presence of a PdZn alloy in XRD (Fig. S11†). Additionally, cyclic and long-term reactions demonstrated the stability of both catalysts, with no signs of decreasing CO_2_ conversion observed over multiple reaction cycles or for lower reduction temperatures (Fig. 4E and S17†).

For MeOH production over Cu/ZnO catalysts, a formate pathway has been proposed in the literature.^86^ A DFT study by Grabow and Mavrikakis^87^ demonstrated that CO_2_ hydrogenation over Cu/ZnO occurs *via* the formate pathway (
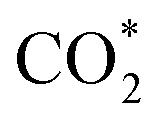
 → HCOO* → HCOOH* → 
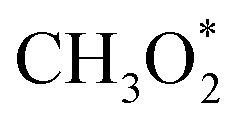
 → CH_2_O* → CH_3_O* → CH_3_OH*). Meanwhile, MeOH production from CO follows the sequence CO* → HCO* → CH_2_O* → CH_3_O* → CH_3_OH*. Nevertheless, it is well documented in the literature that methanation, particularly over Pd-based bimetallic catalysts (*e.g.*, CuPd), can follow either the formate or the reverse-water gas shift (RWGS) pathway.^88^ Future studies employing *in situ* or *operando* techniques may provide confirmation of these intermediates, although the mechanism is still controversially discussed.^84^

## Conclusions

Not unexpectedly, the hydrogenation of CO_2_ or CO to CH_4_ and/or MeOH with catalysts of Cu, Pd, and PdCu NPs supported on ZnO or graphene has shown promise for reducing CO_2_ emissions. Through comprehensive characterization involving various techniques such as HRTEM, EDX, HAADF-STEM, XRD, H_2_-TPR, H_2_-TPD, and D_2_O-TPD, as well as CO_2_/CO hydrogenation kinetics, it was observed that lower Cu or Pd loadings (5%) supported on ZnO or graphene favored CH_4_ production from 300 to 500 °C and 20 bar pressure. CO was the major byproduct, indicating the occurrence of the reverse water gas shift (RWGS) reaction. The addition of the noble metal Pd to Cu had only a minor effect on performance. In contrast, higher Cu content (90%) strongly increased MeOH production in both CO_2_ and CO hydrogenation at high pressure (20 bar).

CO_2_ hydrogenation at ambient pressure (1 bar) and high temperature (300–500 °C) on both Cu and Pd on ZnO produced CO as the major product, corroborating that high pressure is essential for CH_4_/MeOH production.

For CO hydrogenation at 400 to 500 °C, CO conversion was at least 40% higher than CO_2_ conversion, with CH_4_ and CO_2_ identified as the main products. Again, utilizing 90% Cu supported on ZnO led to an enhanced CO conversion of 14%, with a MeOH yield reaching 10% and a CO_2_ yield reaching 4.3% at 230 °C.

The findings underline the significance of catalyst (NP) composition, affected by the corresponding synthesis methodologies, for optimizing CO_2_ and CO hydrogenation processes for efficient methane or methanol production. This offers useful insights for advancing sustainable energy solutions.

## Experimental

### Synthesis of catalysts

The bimetallic catalysts were synthesized employing two distinct methods. For the first method (Fig. 1A), Cu was initially deposited on the support, followed by the addition of palladium salt to initiate a redox reaction and form alloyed NPs. The synthesis was conducted on both ZnO powder and graphene, with a total metal loading of 5 wt% and a Pd/Cu ratio of 2 : 3. Copper acetate monohydrate (0.19 g) was dissolved in distilled water under stirring, followed by the addition of 1.90 g ZnO (or graphene nanoplatelets), and the suspension was stirred overnight. The mixture was then heated to 130 °C to evaporate the water, and the solid was dried for 2 hours at 120 °C. Subsequently, the catalyst was calcined at 500 °C for 2 hours in air and then reduced with H_2_ at 350 °C for 2 hours. In a separate step, a solution of palladium acetate in water was prepared, and the reduced copper catalyst was added to the solution under a flow of nitrogen. After evaporating the water and drying, the catalyst was calcined at 500 °C for 2 hours before reduction in the reactor prior to kinetic measurements. The prepared catalysts are named “CuPd/ZnO-r” and “CuPd/Gr-r”.

In the second method (Fig. 1A), 1-hexadecylamine (HDA) capped Pd–Cu nanoparticles were synthesized and then dissolved in toluene for impregnation on ZnO. The metal loading was 5 wt%, with a Pd : Cu ratio of 2 : 3. In a round-bottom flask equipped with a magnetic stirrer, refluxing device, and nitrogen purging source, 80 mL of ethylene glycol was cooled with an ice bath and degassed for ten minutes under a stream of nitrogen. Copper acetate hydrate (0.95 g) and palladium acetate (0.43 g) were added to the flask, followed by the addition of 4.8 g of HDA to the ethylene glycol solution kept in the ice bath. The mixture was heated to 60 °C until HDA dissolved, resulting in a color change from blue to green. The solution was then heated to 160 °C and maintained at this temperature overnight. The color of the solution changed from deep blue/violet to green, then blue, and finally black, with a red layer, likely Cu_2_O, deposited on the inner wall of the flask. After cooling to room temperature, black NPs were separated by centrifugation and washed three times with ethanol to remove excess HDA and ethylene glycol. The NPs were dried overnight, resulting in a yield of approximately 58.7%. Subsequently, the nanoparticles were deposited on ZnO. The required amount of NPs for a metal loading of 5 wt% was calculated, and 0.1 g of NPs was suspended in toluene with 1.89 g of ZnO. After removing all toluene under vigorous stirring at about 150 °C, the precipitate was dried overnight at 125 °C, followed by calcination in air at 500 °C for 2 hours to completely remove HDA. The reduction of metal oxides was performed in the reactor before conducting kinetic measurements. The resulting catalyst weighed 1.77 g, representing a yield of 88.5%, named “CuPd/ZnO-p”.

Using a co-precipitation method,^61^ copper nitrate (90%) and Zn nitrates (10%) were reduced with aqueous Na_2_CO_3_, dried, and calcined at 350 °C to obtain the “90Cu/ZnO-c” catalyst (Fig. 1A).

The monometallic Cu and Pd catalysts were prepared *via* the wet-impregnation (“-i”) method of a ZnO support. Each catalyst was synthesized by weighing 0.32 g of copper acetate monohydrate or 0.21 g of palladium acetate. The metal salt was then suspended in 30 mL toluene in a flask, ensuring complete dissolution by adding a few drops of ethanol. Subsequently, 1.90 g of ZnO powder was added, and the suspension was stirred overnight at room temperature. The solvent was evaporated by heating the mixture to 150 °C, and the resulting solid was dried for 2 hours in an oven at 120 °C. The calcination of the catalysts was performed in air at 500 °C for 2 hours, using a heating rate of 10 °C min^−1^. This calcination step was essential for removing the acetate from the catalyst and converting the metals into oxides. Prior to kinetic measurements, the catalysts underwent reduction in the reactor. The reduction was carried out using a 10 vol% hydrogen/He flow at three different temperatures: 200 °C, 300 °C, and 500 °C. The prepared catalysts were named “Cu/ZnO-i” and “Pd/ZnO-i”.

### Characterization of catalysts

The morphology and crystal structure of the catalysts were analyzed using an FEI TECNAI G2 F20™ at the University Service Center for Transmission Electron Microscopy (USTEM) at TU Wien. The microscope was equipped with a field emission gun (X-FEG) operated at an acceleration voltage of 200 kV. Additionally, an energy dispersive X-ray (EDX) silicon drift detector (SSD) (EDAX Apollo XLT SSD™) was incorporated into the TEM. Catalyst samples were loaded onto a carbon-coated Cu grid and then inserted into the TEM's inlet system using a single tilt holder. Various TEM images, including high-angle annular dark field (HAADF) and high-resolution (HR) TEM images, were recorded for each catalyst both before and after the reaction. Structural alterations during the reaction were discerned through image comparison, with the high resolution of HRTEM images allowing for precise measurement of lattice planes to identify different phases. Additionally, energy dispersive X-ray spectroscopy (EDX) and electron energy loss spectroscopy (EELS) measurements were conducted to investigate alloy formation. Particle size and distribution were analyzed using DigitalMicrograph software (Gatan™).

The XRD measurements were primarily carried out to elucidate the atomic structure of various crystalline phases (metals, alloys, and oxides). Diffractograms were acquired using a PANanalytical X'Pert Pro™ Bragg–Brentano™ powder diffractometer at the X-ray Center of TU Wien. Cu Kα with a wavelength of 1.54 Å served as the radiation source. A small amount of each catalyst (including calcined catalysts and those subjected to three different reduction temperatures) was applied to a silicon wafer (Si (1 1 1) layer) fixed to a sample holder. The position (2*θ* angle) of the measured reflexes was compared with diffractograms from a database (COD Crystallography Open Database™) to identify crystalline phases. Rietveld refinement was employed to quantify the different compounds present in the diffractograms. This technique utilizes the least squares method to fit a theoretical line profile to the observed reflexes until a satisfactory match is achieved. Furthermore, Rietveld refinement facilitated the calculation of the crystallite size of both the support and the metal particles (using HighScore Plus 4.1 – PANalytical software™).

Temperature programmed reduction (TPR) served as a standard method for gathering insights into the reducibility of a material. The H_2_ TPR analysis was conducted in a continuous fixed bed quartz tube reactor. Approximately 50 mg of catalyst was loaded into the reactor tube, which was then positioned in a heating furnace. Gas flows of argon and H_2_ (the reducing gas) were carefully regulated using calibrated mass flow controllers. A total flow of 100 mL min^−1^ with 10 vol% H_2_ in Ar was passed through the sample. During the experiment, the furnace underwent heating from room temperature to 500 °C at a rate of 10 °C min^−1^. The quartz tube reactor was linked to a quadrupole mass spectrometer (Balzers Prisma™), which recorded the mass signals of H_2_ (*m*/*z* = 2) and H_2_O (*m*/*z* = 18) over time as a function of temperature. These experiments were carried out for both synthesized catalysts and the pure ZnO support.

Temperature programmed desorption (TPD) provided insights into the binding energy of absorbed molecules. The experiments were conducted within a solid quartz tube reactor in conjunction with a quadrupole mass spectrometer (Balzers Prisma™). Each catalyst, weighing 50 mg, was fixed with quartz wool and loaded into the reactor tube. To facilitate the adsorption of gas molecules on the surface, the catalyst was exposed to a gas mixture containing probe molecules such as H_2_ or D_2_O (100 or 10 mbar gas pressure, respectively). The Redhead approximation (eqn (1))^70^ can be applied for calculating the binding energy of molecules on the catalyst's surface:1
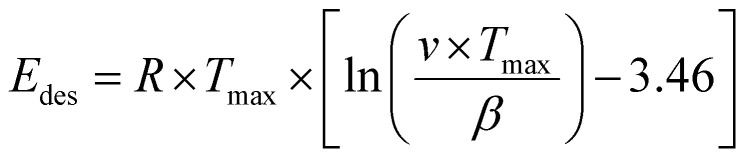
*E*_des_ is the activation energy of desorption, *T*_max_ is the temperature at the peak maximum, *β* is the heating rate (d*T*/d*t*), *n* is the order of desorption and *ν* is the frequency factor.

In general, the Redhead equation is valid for first-order desorption (single step desorption). As an approximation, eqn (1) was used with a frequency factor of *v* = 10^13^ s^−1^.

Moreover, D_2_O TPD of ZnO was carried out after reduction at various temperatures, and *m*/*z* = 4 signals were recorded. This experiment provided insights into possible vacancies within the support. Due to the interaction of D_2_O with the surface defects of ZnO, D_2_ was formed and detected by the mass spectrometer (*m*/*z* = 4). The same preparation and measurement steps as those used for H_2_ TPD were applied, with a partial pressure of 10 mbar D_2_O utilized for adsorption.

### CO_2_ and CO hydrogenation

The kinetic measurements were conducted using a PID Eng&Tech Microactivity Effi Reactor™, a 30 cm long stainless-steel tubular reactor with an internal diameter of 9.91 mm, in a temperature range of 25 to 800 °C and pressures up to 20 bar. Feed gases including H_2_, O_2_, CO, CO_2_, and He were introduced into the reactor, with flow rates regulated by calibrated mass flow controllers. Online product analysis was performed *via* a micro-GC system (Inficon™; 300 Micro GC, runtime < 2 min), equipped with a molecular sieve, a plotQ column™ and a Thermal Conductivity Detector (TCD). The molecular sieve effectively separated small molecules such as H_2_, O_2_, and N_2_, while the plotQ column facilitated the separation of carbon-containing compounds. Kinetic measurements were conducted for CO_2_ and CO hydrogenation at 20 bar (or ambient pressure) and various temperatures ranging from 230 to 500 °C. Each temperature step was held constant for 8 hours, with average values taken for further calculations. This duration allowed the catalyst to reach a quasi-stationary state, minimizing the effects of transient behaviour. The total gas flow was maintained at 10 mL min^−1^ (corresponding to a flow of 6.81 × 10^−6^ mol s^−1^), comprising 5% CO_2_ or CO and 20% H_2_ in helium. Following each reaction period at a specific temperature, a reactivation treatment was applied. For this, the catalyst underwent oxidation with 10% O_2_ in He at 500 °C to remove carbonaceous deposits, followed by reduction with 10% H_2_ in He.

The TCD was calibrated prior to the experiments using standard gas mixtures to ensure accurate quantification of the detected species. Calibration curves were generated by plotting the peak areas against the known concentrations of the standards (CO_2_, CO, and H_2_, respectively). Linear regression was used to determine the relationship between the peak area and concentration. During the experiments, the peak areas corresponding to the different reactants and products were recorded. These peak areas were then used to determine the concentrations of the molecules present. The calibrated peak areas from GC chromatograms were then utilized for the calculation of, *e.g.*, the conversion_CO_2__ (%), as given in eqn (2),2
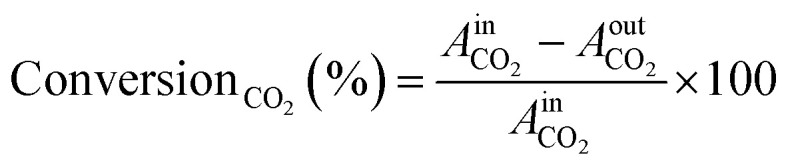
where 
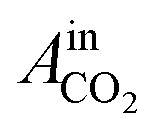
 = peak area of CO_2_ entering the reactor and 
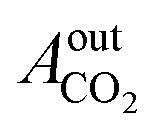
 = peak area of CO_2_ exiting the reactor.

Analogously, for CO hydrogenation, the CO GC peak areas were utilized for the calculation of the CO conversion.

The selectivity of the catalysts for producing MeOH selectivity_MeOH_ (%) was determined using eqn (3),3
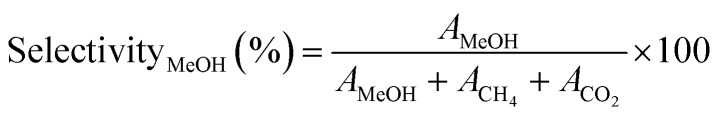
where *A*_MeOH_ = peak area of MeOH, *A*_CH_4__ = peak area of CH_4_ and *A*_CO_2__ = peak area of CO_2_.

Similarly, the CH_4_ selectivity_CH_4__ (%) was determined using eqn (4)4
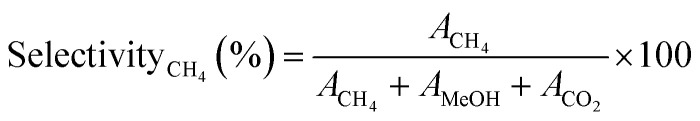


With conversion (eqn (2)) and selectivity (eqn (3) or (4)), the yield of MeOH, CH_4_, or CO can be calculated using eqn (5)–(7),5
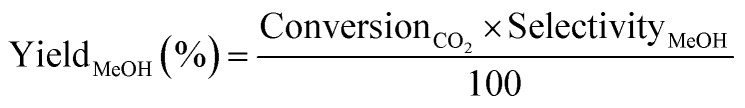
6
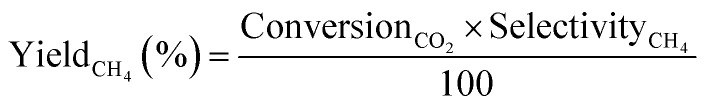
7
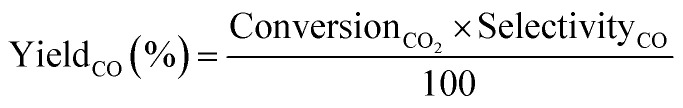


For CO hydrogenation, yields were calculated analogously.

For the Gas Hourly Space Velocity (GHSV), Residence Time (*τ*), and Space Time Yield (STY) calculations, refer to Note 1 of the ESI.†

## Data availability

The data supporting this article have been included as part of the ESI.†

## Author contributions

QM: conceptualization, methodology, validation, software, formal analysis, investigation, data curation, writing – original draft, and writing – review & editing. KD: conceptualization, methodology, validation, and formal analysis. JS: data curation and writing – review & editing. MH: methodology, validation, formal analysis, investigation, and writing – review & editing. KLK, TS, SL, AA, and JN: formal analysis, data curation, validation, and writing – review & editing. GR: conceptualization, validation, resources, writing – review & editing, and supervision.

## Conflicts of interest

There are no conflicts of interest to declare.

## Supplementary Material

SU-002-D4SU00339J-s001
