# Supplementary material for: Bimetallic CuPd nanoparticles supported on ZnO or graphene for CO2 and CO conversion to methane and methanol
Source: RSC Sustain. 2024 Sep 4;2(11):3276–88. doi: 10.1039/d4su00339j (PMC11418587; doi:10.1039/d4su00339j)
Supplement: SU-002-D4SU00339J-s001 [file SU-002-D4SU00339J-s001.pdf]

Supplementary Information (SI) for RSC Sustainability.
This journal is © The Royal Society of Chemistry 2024

Electronic Supplementary Information (ESI) for

Bimetallic CuPd Nanoparticles Supported on ZnO or Graphene for CO_2 and CO Conversion to Methane and Methanol

Qaisar Maqbool[a,‡], Klaus Dobrezberger[a,‡], Julian Stropp[a], Martin Huber[a], Karl-Leopold Kontrus[a], Anna Aspalter[a], Julie Neuhauser[a], Thomas Schachinger[b], Stefan Löffler[b], and Günther Rupprechter[a]*

[a]Institute of Materials Chemistry, TU Wien, Getreidemarkt 9/BC, A-1060 Vienna, Austria.
[b]University Service Center for Transmission Electron Microscopy, TU Wien, Stadionallee 2/057-02, 1020 Vienna, Austria.

[‡]Authors with equal contributions
*Correspondence: guenther.rupprechter@tuwien.ac.at

CuPd/ZnO-r (after CO_2 hydrogenation)

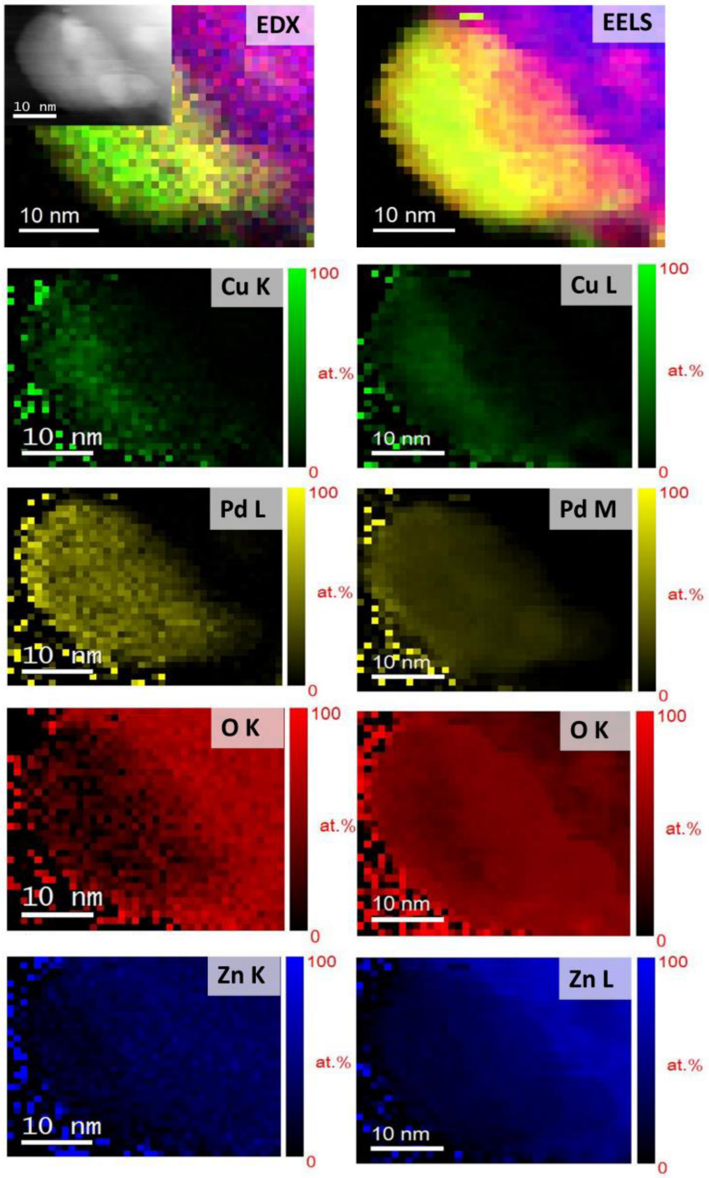

Supplementary Figure S1. HAADF-STEM image with EDX and EELS mappings of CuPd/ZnO-r catalyst after CO_2 hydrogenation reaction showing the distribution of oxygen, palladium, copper, and zinc.

CuPd/ZnO-r (after CO hydrogenation)

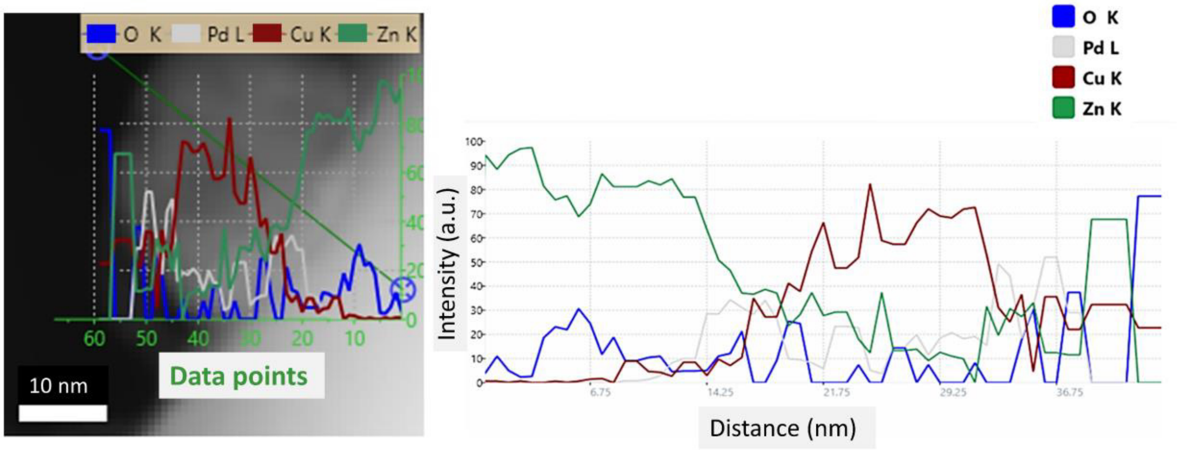

Supplementary Figure S2. HAADF-STEM image with an EDX line scan of CuPd/ZnO-r catalyst after CO hydrogenation reaction showing the distribution of oxygen, palladium, copper, and zinc.

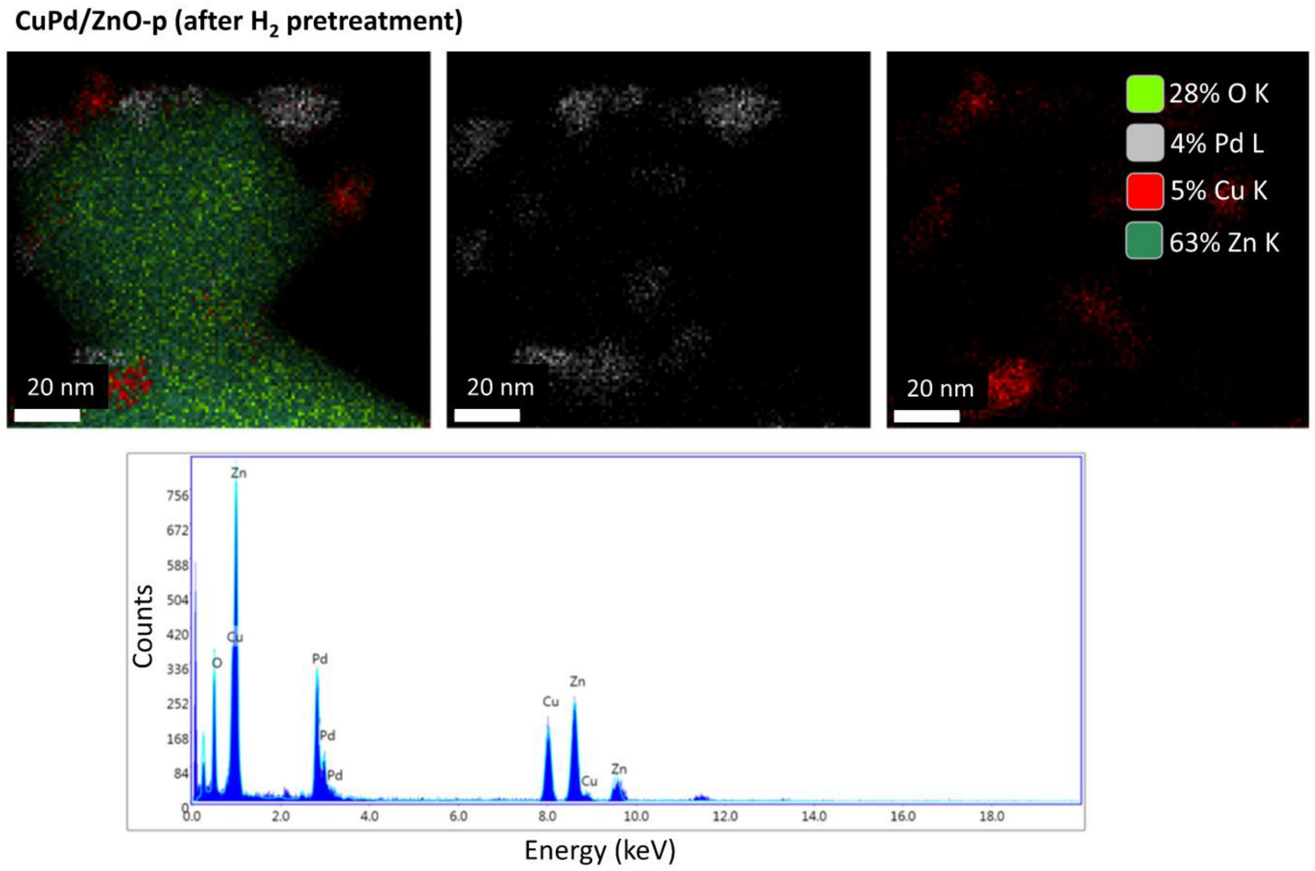

Supplementary Figure S3. EDX mapping and EDX spectrum of CuPd@ZnO-p catalyst after H_2-reduction at 500 °C, showing the distribution of oxygen, palladium, copper, and zinc.

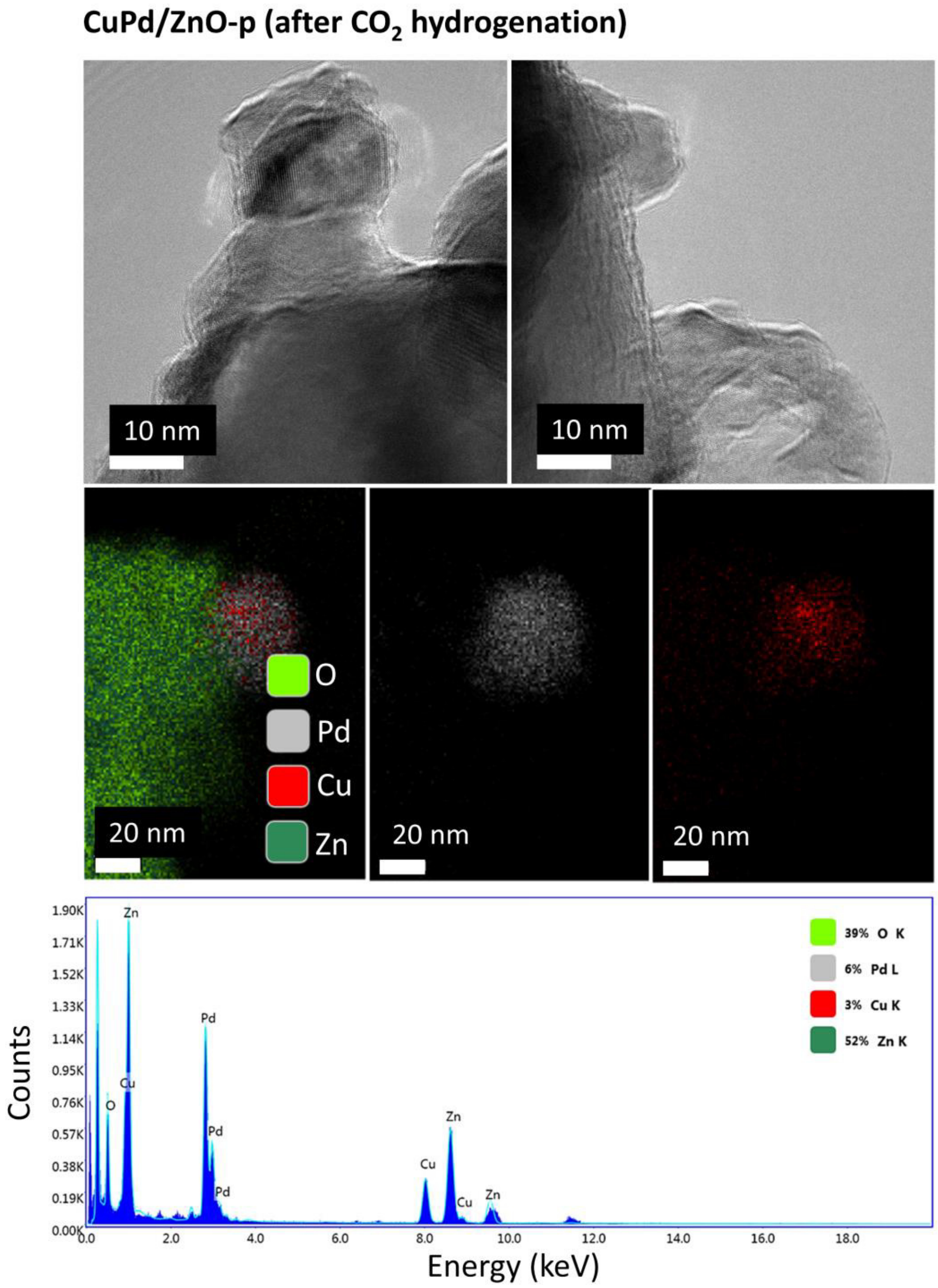

Supplementary Figure S4. TEM images with an EDX mapping and EDX spectrum of CuPd/ZnO-p catalyst after CO_2 hydrogenation reaction showing the distribution of oxygen, palladium, copper, and zinc.

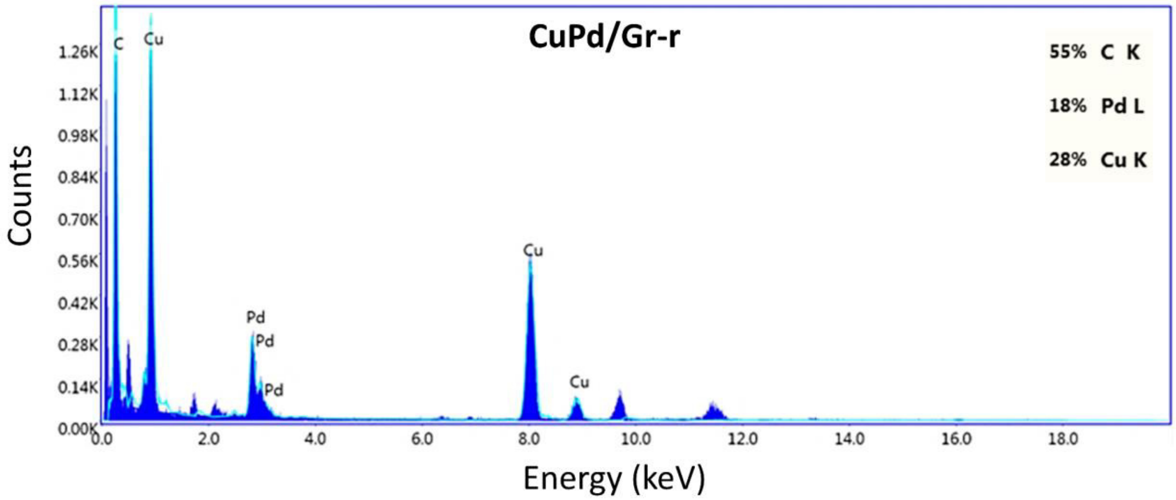

Supplementary Figure S5. EDX spectra of H_2-reduced CuPd/Gr-r, showing the percentage of carbon, palladium, and copper.

CuPd/Gr-r (after CO_2 hydrogenation)

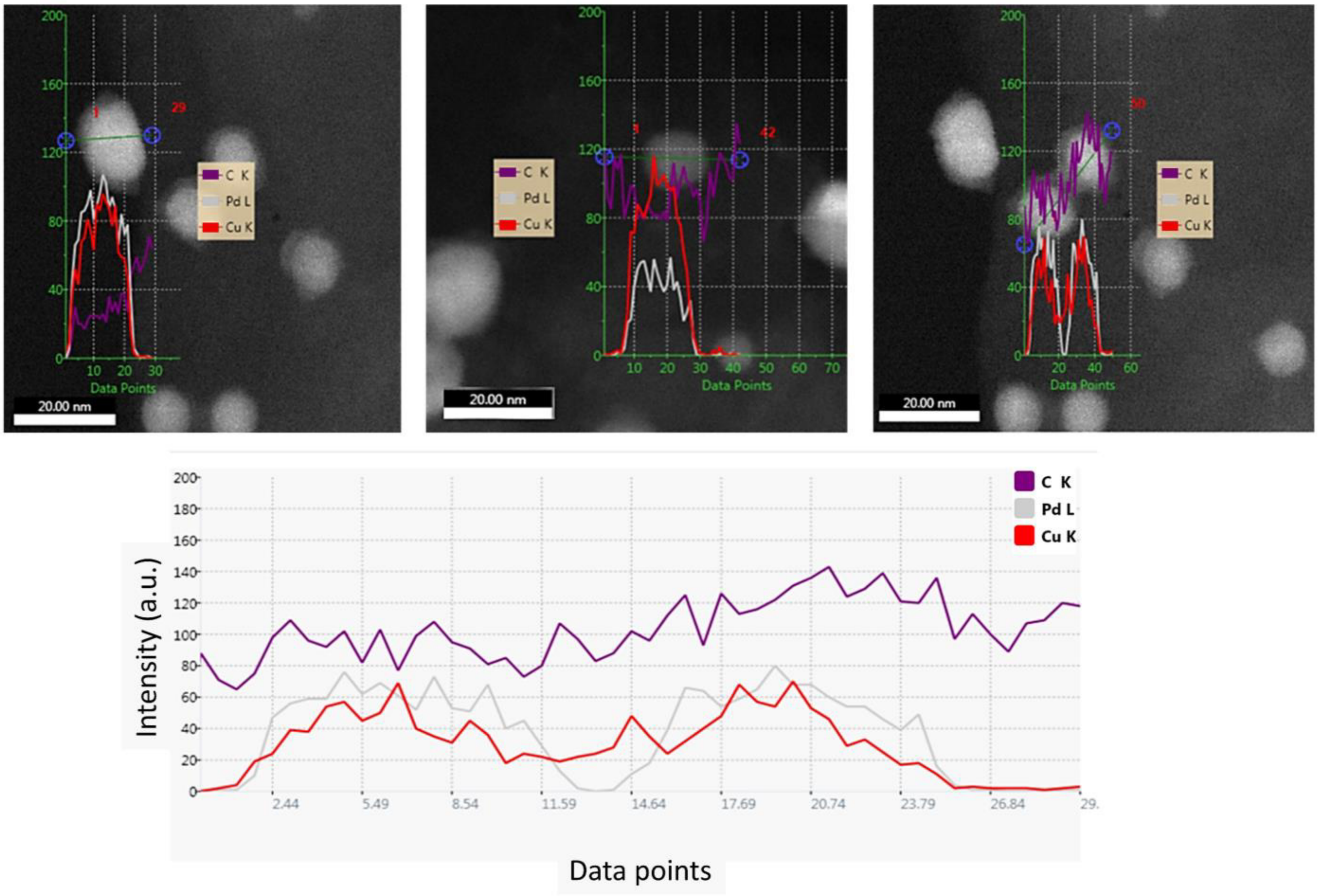

Supplementary Figure S6. HAADF-STEM images with line scans of CuPd/Gr-r catalyst after CO_2 hydrogenation reaction showing the distribution of carbon, palladium, and copper.

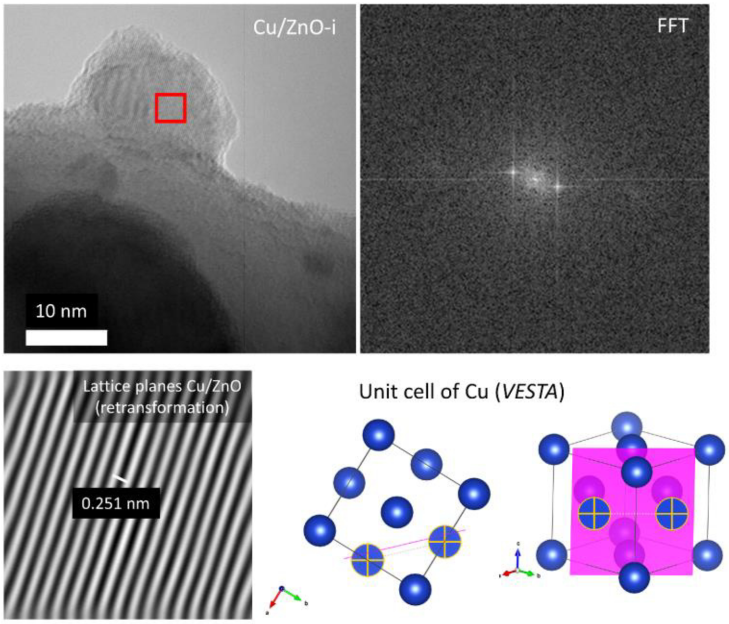

Supplementary Figure S7. HRTEM of Cu/ZnO-i catalyst after reduction in H_2 (500 °C) depicts the ZnO support with a single Cu NP situated on the surface, FFT, inverse FFT showing Cu/ZnO lattice planes. Unit cell dimensions of Cu obtained from VESTA®.

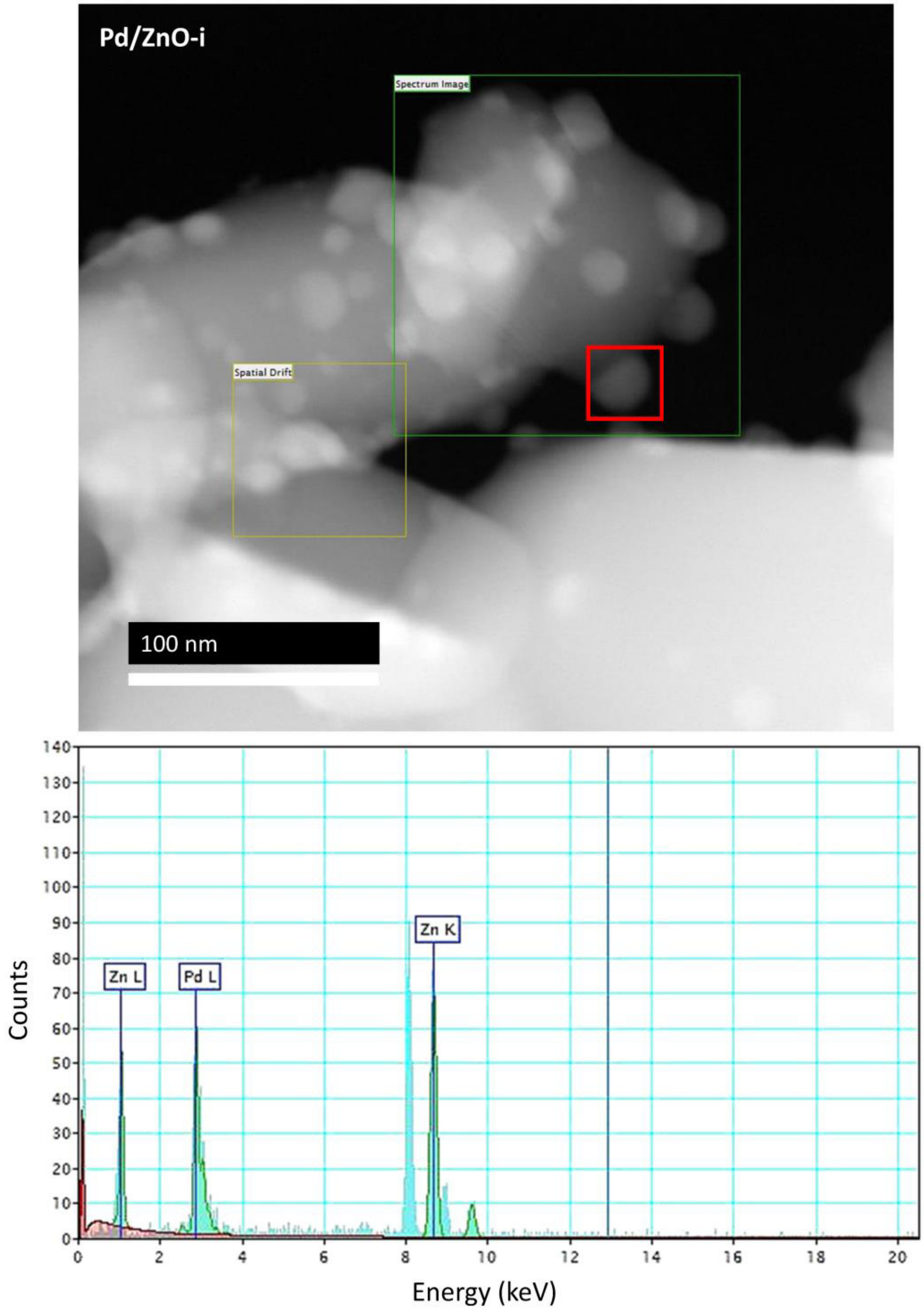

Supplementary Figure S8. HAADF-STEM image of Pd/ZnO-i after reduction at 500 °C, also showing EDX spectrum from marked area (red box).

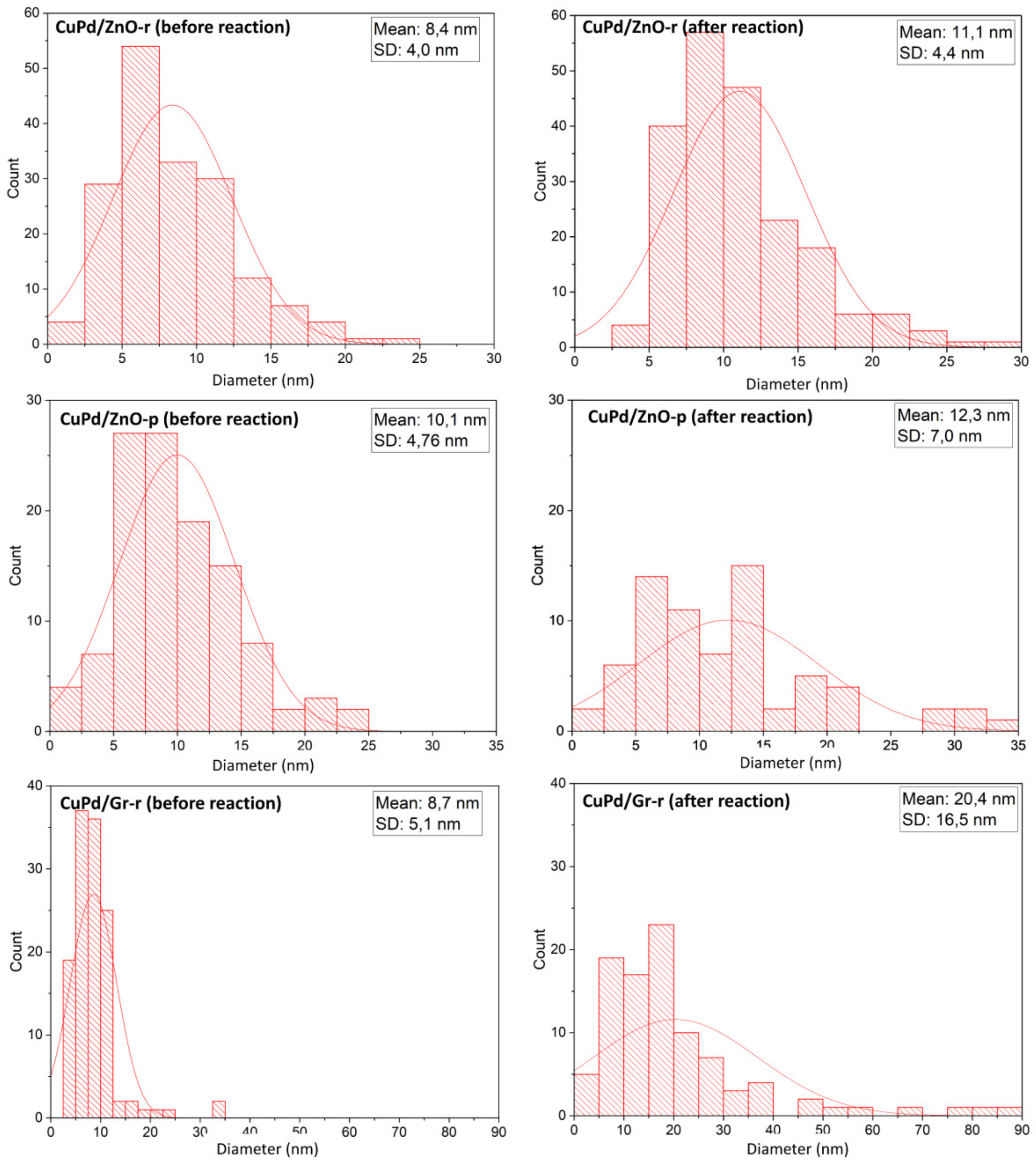

Supplementary Figure S9. Change in NPs (CuPd/ZnO-r, CuPd/ZnO-p, and CuPd/Gr-r) mean size due to sintering at high temperature and pressure.

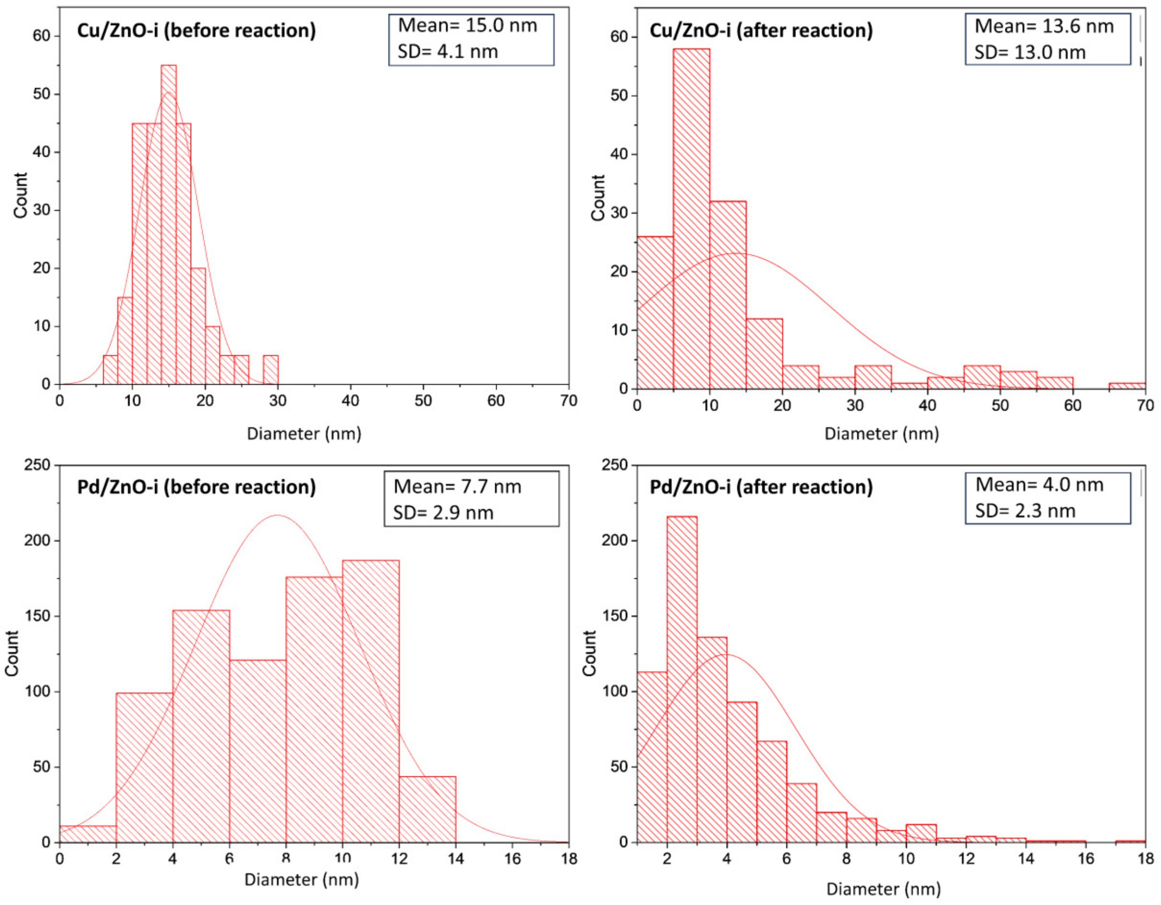

Supplementary Figure S10. Change in NPs (Cu/ZnO-i and Pd/ZnO-i) mean size due to sintering at high temperature and pressure.

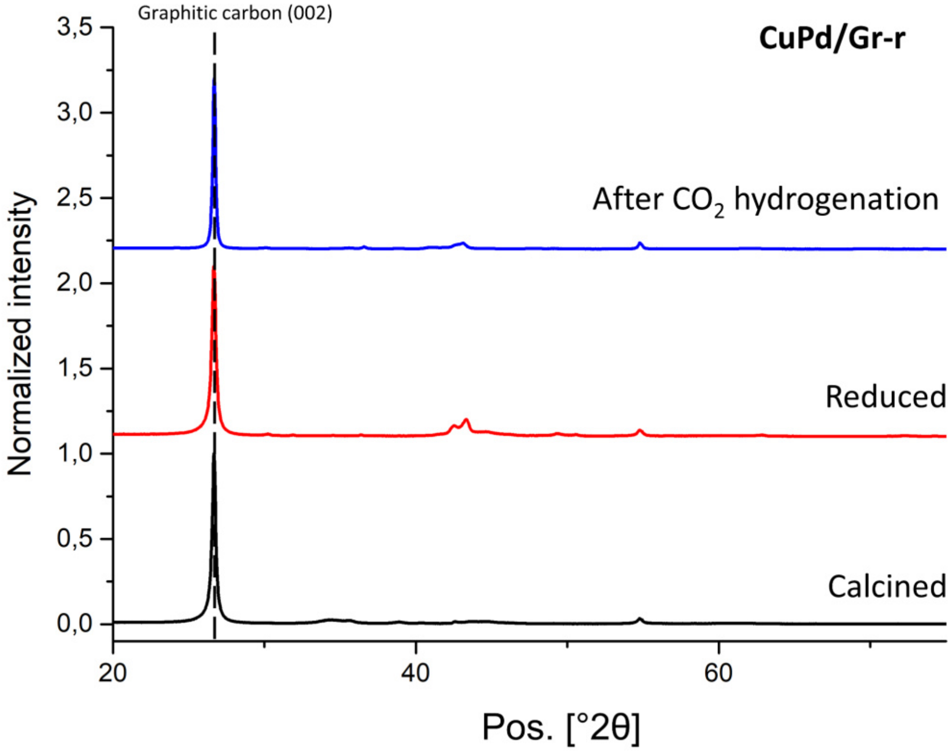

Supplementary Figure S11. XRD patterns of the CuPd/Gr-r catalyst: calcined (500°C), H_2-reduced and after CO_2 hydrogenation.

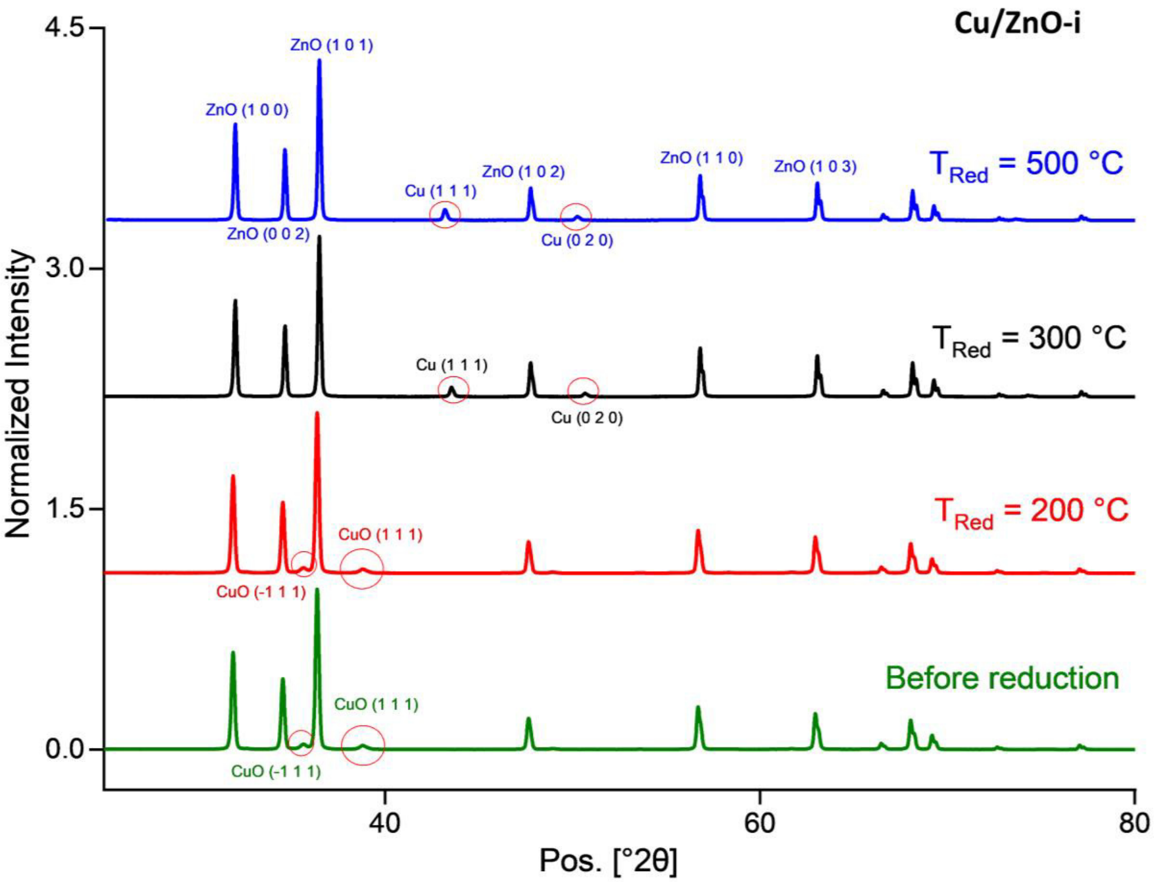

Supplementary Figure S12. XRD patterns of the calcined (500°C), and H_2-reduced (T=200-500 °C) Cu/ZnO-i catalyst.

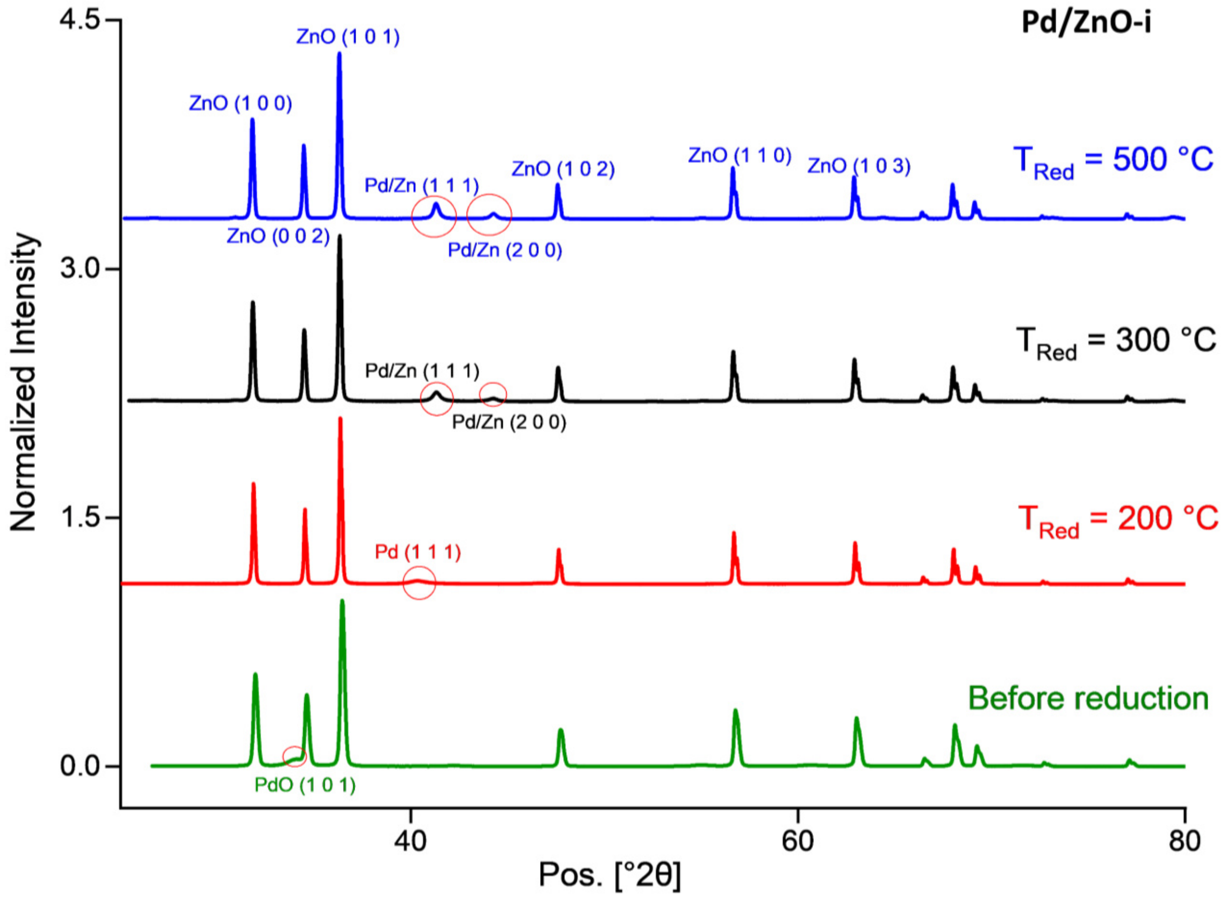

Supplementary Figure S13. XRD patterns of the calcined (500°C), and H_2-reduced (T=200-500 °C) Pd/ZnO-i catalyst.

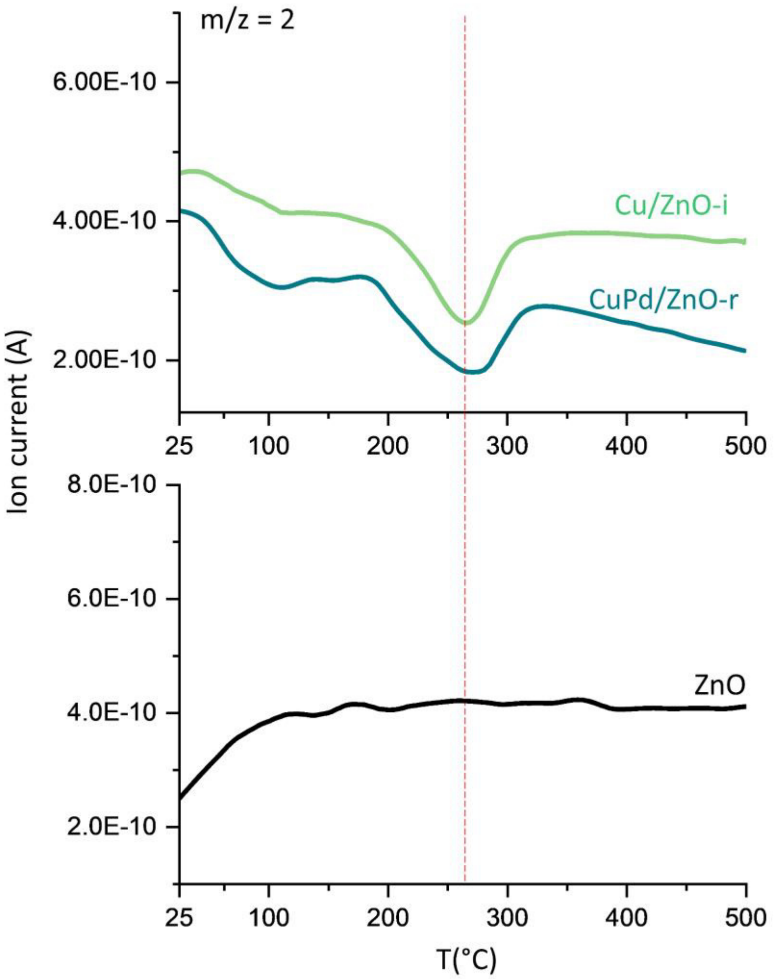

Supplementary Figure S14. H_2-temperature programme reduction (TPR) profile of calcined (500°C) Cu/ZnO-i, CuPd/ZnO-r catalyst, and ZnO support.

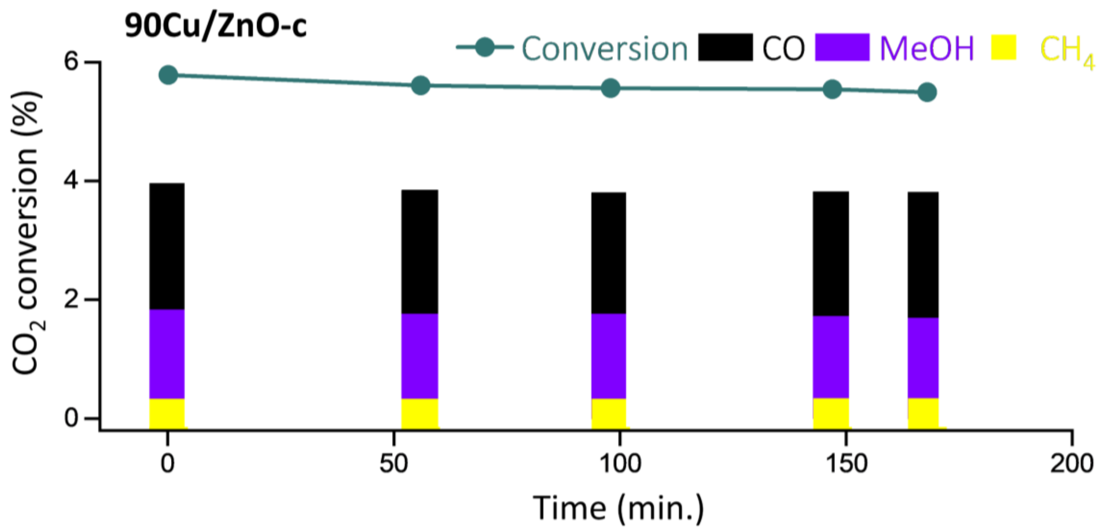

Supplementary Figure S15. CO_2 hydrogenation to MeOH (%) and CO (%) by 90Cu/ZnO-c catalysts at p = 20 bar and T= 230°C.

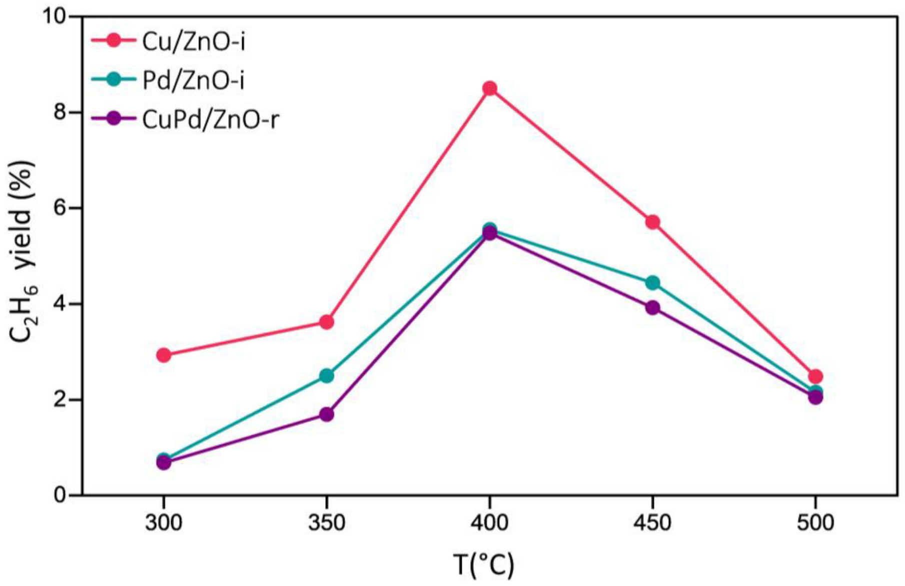

Supplementary Figure S16. Ethane as byproduct (%) of CO hydrogenation on Cu/ZnO-i, Pd/ZnO-i, and CuPd/ZnO-r catalysts.

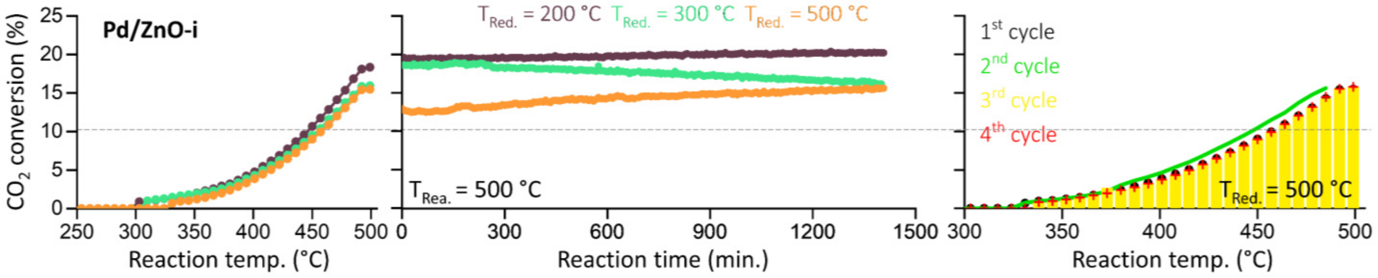

Supplementary Figure S17. CO_2 conversion to CO (%) by Pd/ZnO-i catalyst reduced at different temperatures, at p = 1 bar and T= 300-500°C, for up to 24 hours., also showing CO_2 conversion to CO (%) over multiple cycles (for Pd/ZnO-i catalyst reduced at 500°C).

Table S1. Composition analysis of CuPd/Gr-r with Vegard's rule: d (lattice spacing); hkl (Miller indices); a (lattice parameter); p (atomic percentage).

d	hkl	a (nm)	p(Cu)	p(Pd)
0,212	111	0,367	0,69	0,31
0,211	111	0,365	0,75	0,25
0,216	111	0,374	0,46	0,54
0,224	111	0,388	0,00	1,00

Table S2. Quantification result of EDX spectra of CuPd/Gr-r.

Element	Weight%	Atomic%
C K	20,96	61,93
Pd L	27,07	9,03
Cu K	51,97	29,04

Supplementary note 1:

Gas Hourly Space Velocity (GHSV) and Residence Time (τ)

To measure the interaction between the reactants (e.g. CO_2/H_2 or CO/H_2) and the catalyst bed in the reactor, it is necessary to calculate the gas hourly space velocity (GHSV). The GHSV describes the volume of reactants that passes through the catalyst bed per hour. Therefore, the total volumetric flow of the reactants, as well as the volume of the catalyst bed, are required for its calculation, as shown in the equation below,

$$\mathrm{GHSV} = \frac{\dot{\mathrm{V}}_{\mathrm{Reactants}}}{\mathrm{V}_{\mathrm{Catalyst}}} = \frac{\dot{\mathrm{V}}_{\mathrm{Reactants}}}{\pi \times \mathrm{r}^2 \times \mathrm{l}}$$

GHSV = Gas Hourly Space Velocity (h^{-1})

$\dot{V}_{Reactants}$ = Volumetric flow rate of reactants (0.15 L/h)

$V_{Catalyst}$ = Volume of catalytic bed (9.1×10^{-5} L)

r = internal radius of reactor (4.55×10^{-2} dm)

l = length of catalytic bed (1.40×10^{-2} dm)

GHSV = 1648.4 h^{-1}

Moreover, the reciprocal value of the GHSV is the residence time (τ), so that

$$\tau = \frac{1}{GHSV} \times 3600$$

meaning that a reactant molecule spends 2.18 seconds over the catalyst bed before leaving the reactor.

Space-Time Yield (STY)

The space-time yield (STY) is another parameter used to characterize the performance of a catalyst. To determine this, the volume of the reagent gas is calculated according to equation **(1)**, using the residence time and the gas flow rate. The molar amount of the product, assuming that it equals that of the reagent gas, is determined by rearranging the universal gas equation **(2)** and then converting it into grams **(3)**. The STY values are finally obtained by inserting the measured CH_4 or MeOH yield into equation **(4)**.

$$\mathrm{V}_{CO_2\ \mathrm{or\ CO}} = \dot{\mathrm{V}}_1 \cdot \tau \qquad \textbf{(1)}$$

V_1 = total gas flow of CO_2 or CO (0.5 mL/min = 0.03 L/h)

t = residence time (τ) (0.0006 h)

$V_{CO_2\ \mathrm{or\ CO}}$ = volume of CO_2 or CO (0.000018 L)

$$n_{CO_2\ or\ CO} = \frac{p \cdot \mathrm{V}_{CO_2 \text{ or CO}}}{\mathrm{R} \cdot T} = n_p \qquad \textbf{(2)}$$

p = reaction pressure (20 bar)

R = universal gas constant (0.083143 L·bar·mol^{-1}·K^{-1})

T = optimum reaction temperature (CH_4 - 773.15 K, CH_3OH – 503.15 K)

$n_{CO_2\ or\ CO}$ = amount of CO_2 or CO (CH_4 - 0.0000056 mol, CH_3OH – 0.0000086 mol)

n_p = maximum amount of product (CH_4 - 0.0000056 mol, CH_3OH – 0.0000086 mol)

$$m_p = n_p \cdot M_p \qquad \textbf{(3)}$$

M_p = molar mass of product (16.04 g/mol for CH_4 or 32.04 g/mol for CH_3OH)

m_p = maximum amount of product of product (CH_4 - 0.00009 g, CH_3OH – 0.00028 g)

$$STY = \frac{Yield \cdot m_p}{100 \cdot m_{cat} \cdot \tau} \qquad \textbf{(4)}$$

Yield = measured yield (CH_4 : CO_2 hydrogenation - 16.14 % or CO hydrogenation - 50.88 %;
CH_3OH: CO_2 hydrogenation – 1.82 % or CO hydrogenation – 10.47 %)

m_{cat} = mass of catalyst (0.1 g)

STY= space-time yield: CH_4: CO_2 hydrogenation - 0.2416 g_{CH_4}·g_{cat}^{-1}·h^{-1}, CO hydrogenation – 0.7618 g_{CH_4}·g_{cat}^{-1}·h^{-1};
CH_3OH: CO_2 hydrogenation - 0.0836 g_{MeOH}·g_{cat}^{-1}·h^{-1}, CO hydrogenation – 0.4811 g_{MeOH}·g_{cat}^{-1}·h^{-1} .
